# Coupled fish-hydrogeomorphic responses to urbanization in streams of Columbus, Ohio, USA

**DOI:** 10.1371/journal.pone.0234303

**Published:** 2020-06-15

**Authors:** Leslie O. Rieck, S. Mažeika P. Sullivan

**Affiliations:** Wilma H. Schiermeier Olentangy River Wetland Research Park, School of Environment and Natural Resources, The Ohio State University, Columbus, Ohio, United States of America; Universitat de Barcelona, SPAIN

## Abstract

Despite a developing literature on urban streams, few studies have addressed the timing and mechanisms of urban-induced stream hydrogeomorphic adjustment on biotic assemblages. Here, we investigated the relationships between urbanization-driven annual changes in fluvial geomorphic characteristics and fish assemblages in 12 headwater streams in the Columbus Metropolitan Area (CMA), Ohio (USA) over 3–5 years. Multiple stream hydrogeomorphic characteristics changed over time including slope (0.1% decrease on average), discharge (39% decrease), and shear stress (29% decrease), some in concert with one another (e.g., slope and shear stress). Species-specific fish associations with hydrogeomorphic associations varied in nature and strength by year and thus were somewhat equivocal. At the assemblage level, we observed a negative relationship between D_50_ (median sediment particle size) and % tolerant individuals as well as a positive trend between incision ratio and % generalists over study years. Study reaches with higher total catchment imperviousness were associated with both finer median sediment size (*R*^*2*^ = 0.19) and lower assemblage diversity (*R*^*2*^ = 0.55). These results contribute to current understanding of the drivers of fish assemblages in urbanizing catchments, and point to urban-induced hydrogeomorphic alterations as one mechanism through which land-use changes influence in-channel characteristics important to aquatic biota.

## Introduction

Fluvial geomorphic features occupy a central role in many patch-dynamics perspectives, where spatial heterogeneity in physical processes and patterns influences habitat, disturbance regimes, and the structure and function of stream ecosystems (e.g., Process Domains Concept [1]; River Ecosystem Synthesis [2]). Within this context, empirical evidence supporting the importance of spatial variability in stream geomorphic characteristics to stream biota and function is growing [3–7]. Although theoretically supported, investigations of the temporal impacts of stream geomorphic characteristics (i.e., the effects of channel adjustment and evolution over time) on ecological structure and function are few and less well resolved [8–10].

Urban development can strongly alter stream geomorphic features, although the changes to channels can vary by region, history of construction, and landscape context [11–13]. Increases in impervious surface area, commonly used to measure the degree of urban development, typically result in decreases in water infiltration and increases in surface runoff during storm events. For instance, increases in runoff of up to 45% have been observed in catchments with high impervious surface cover relative to fully forested catchments [14, 15]. Increased runoff leads to greater frequency of high-flow events (i.e., bankfull flows) that can lead to channel aggradation, widening, or degradation [14, 16, 17]. Active development (e.g., clearing land, construction) in the catchment increases the amount of sediment transported into the channel, and can cause channel aggradation in the short term (months to years, dependent on duration of exposted soil; [16, 18, 19]). Following urban development, flows of higher magnitude and frequency tend to continue as land in the catchment is converted to impervious surfaces or until surfaces are re-vegetated. During this period, channels eventually incise and then widen and sometimes aggrade until the new streamflow regime is accommodated [13, 20, 21].

**Fig 1:**
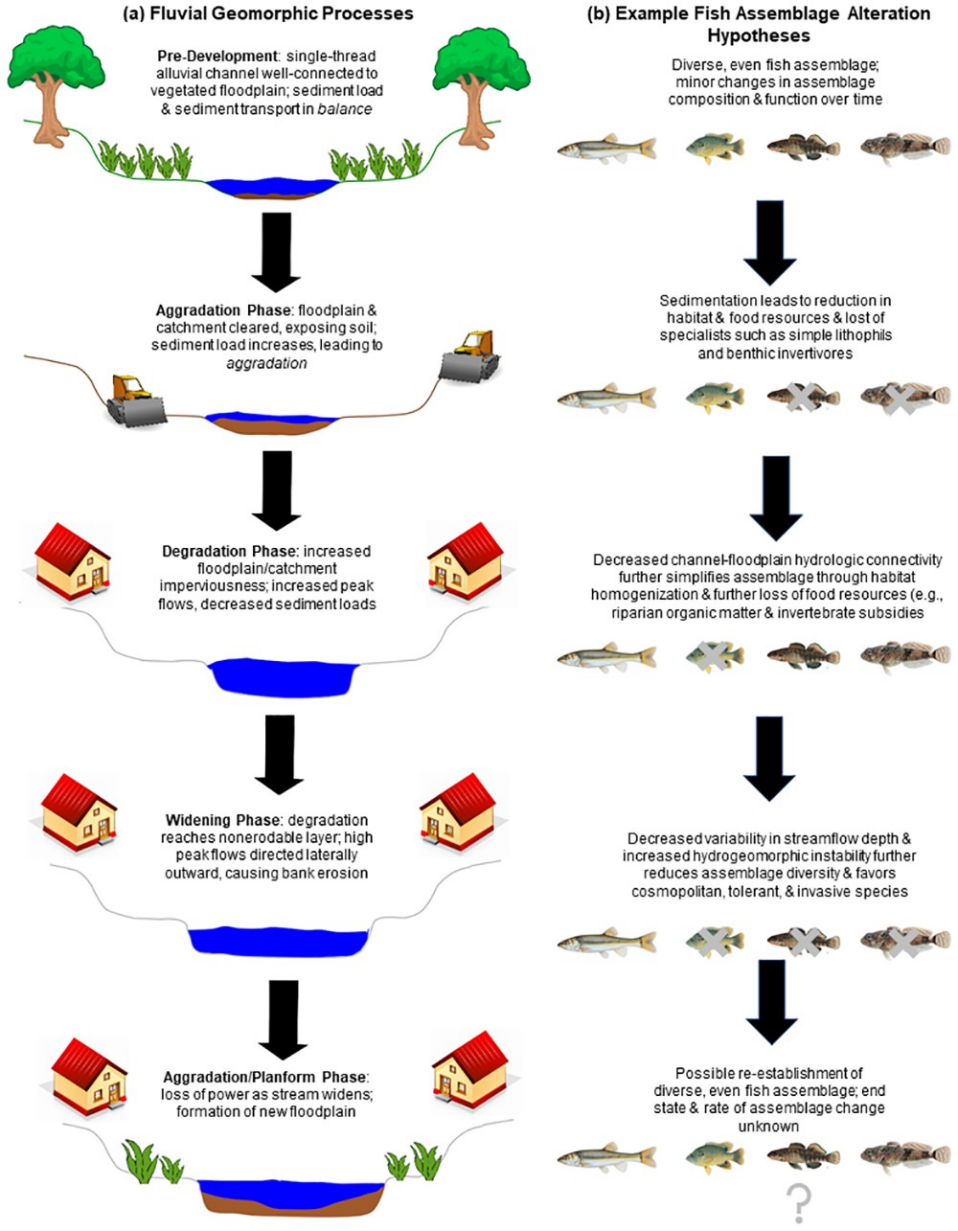
Conceptual depiction of urban stream channel hydrogeomorphic evolution with example fish assemblage alterations. (a) Conceptual depiction of fluvial geomorphic processes commonly observed during different phases of urban development, which can lead to variable responses including reduced sinuosity and increased sediment transport [29, 30]; altered slope, bed and bank materials, and riparian vegetation [31]; and reduced large wood, connectivity to the riparian zone, and retention of organic matter [32, 33]. Note that the three post-development phases (1) do not all necessarily occur and (2) may not proceed in the order depicted. (b) Examples of hypothesized impacts on urban-induced geomorphic alterations on fish assemblages. Fish species (from left to right) are Creek Chub (*Semolitus atromaculatus*), Green Sunfish (*Lepomis cyanellus*), Rainbow Darter (*Etheostoma caeruleum*), and Mottled Sculpin (*Cottus bairdii*).

This series of observed alterations in response to urban-induced disturbance (i.e., pulse disturbances after new construction followed by the long-term press disturbance of sustained urban land use) mirrors Schumm’s [22] five-phase channel adjustment scheme following an alteration in sediment load or streamflow (Fig 1). However, more recent studies have identified that this sequence of channel evolution does not always occur [13, 23, 24]. Not all stream reaches progress along Schumm’s [22] evolution pathway, indicating additional factors (e.g., underlying geology, stormwater infrastructure, bed and bank materials) also influence channel responses to changes in land use. Hawley et al. [25] modified Schumm’s [22] channel evolution model to include the potential for braided channels to occur as a result of urban hydrologic regimes. Connectivity to riparian and floodplain zones is also important to geomorphic processes: access to floodplains may allow some mitigation of high stormflows even in urban streams, decreasing shear stress and, therefore, erosion of streambed and banks [26]. Collectively, channel changes associated with urbanization may disconnect a stream from its riparian zone and floodplain both hydrologically and energetically by limiting or severing the bidirectional exchanges of carbon, organic matter, and prey between stream and riparian zone ([27, 28]; Fig 1).

The effects of fluvial geomorphic shifts on fish assemblages in urban streams are documented in several studies [34–36]. Changes in channel dimensions, altered longitudinal and lateral hydrological connectivity, and shifts in bed materials can contribute to decreases in fish assemblage diversity and evenness in urban streams [37]. For instance, in tropical urban streams, both reduced longitudinal connectivity (i.e., geomorphic and hydrologic connectivity) and increased alteration of bed morphology (e.g., channelization) resulted in reduced relative abundance of native fish species and an increase in relative abundance of non-native species [38]. Urban land cover; high embeddedness, lower slopes, and chemical water-quality variables (e.g., high turbidity) were negatively associated with fish assemblage Index of Biotic Integrity (IBI) scores, abundance, and the relative ratio of endemic species vs. cosmopolitan species in urban areas of the Georgia Piedmont, USA [34].

There is also limited evidence that stream geomorphic adjustments in urban catchments can alter the functional feeding composition. Schwartz et al. [39] found that an incised, channelized stream dominated by run habitat supported fewer insectivorous darter (genus Etheostoma) and sunfish (genus Lepomis) species than the same channel once riffle-pool morphology and bank stability were restored. Streams in developed catchments with heavy silt loads have been shown to exhibit a greater proportion of smaller, more rapidly maturing fish species and a homogenization of functional traits [40]. Peressin and Cetra [41] implicated a loss of substrate quality (i.e., sedimentation, increase in fine substrates), lack of vegetative protection for juvenile fishes and periphyton growth, and decreased bank stability associated with highly urbanized reaches as mechanisms driving observed increases in the relative abundance of omnivores and decreases in the relative abundances of insectivores and periphytivores along a gradient of Brazilian urban stream reaches (e.g., low to high intensity). However, the rate of change in fish assemblages and the response to different levels of urbanization remains unclear.

Understanding how, and over what time scales, urbanization alters stream geomorphic and ecological characteristics, and whether streams can recover from such alterations, is a critical step in further understanding and effectively managing urban streams [30]. In particular, coordinated geomorphic-biotic responses over time are not well represented in the literature (but see e.g., [42, 43]). Here, we investigated coupled fluvial geomorphic-fish assemblage changes over three to five years in a suite of urban, Columbus, Ohio (USA) streams. Owing to the strong associations between reach-scale geomorphology and fish assemblages documented in multiple investigations [5, 8, 44], we hypothesized that fish assemblages would reflect changes in stream geomorphic features. We developed three sets of specific predictions: (1) The relative abundance of fish species in the assemblage would be associated with hydrogeomorphic features such as sinuosity, discharge, incision ratio, and slope owing to species-specific habitat and foraging requirements [39, 40]. (2a) Fish-assemblage diversity and density would decrease in streams with higher rates and magnitudes of geomorphic change (e.g., shifts in substrate size, changes in channel geometry and slope, bank erosion [34, 37, 42]) due largely to losses of benthic invertivores (due to their reliance on diverse benthic invertebrate communities [41], and that (2b) streams that had limited access to their floodplains (e.g., high incision ratios, less frequent overbank flows) would support a higher relative abundance of generalist species due to reduced substrate and habitat quantity and quality [40] (3) Broader-scale impacts of human landscape alteration (e.g., imperviousness in the catchment, time since development) would relate to changes in fish assemblage attributes over time as overarching drivers of hydrogeomorphic, chemical water-quality, and physical-habitat alteration.

## Materials and methods

All research activity involving fish was conducted with approval from the Ohio Department of Natural Resources Collection, Division of Wildlife (Permit 15–49) and was approved by The Ohio State University Animal Care and Use Committee (IACUC Protocols 2008A0161-R1,R2,R3).

### Study system and experimental design

**Fig 2:**
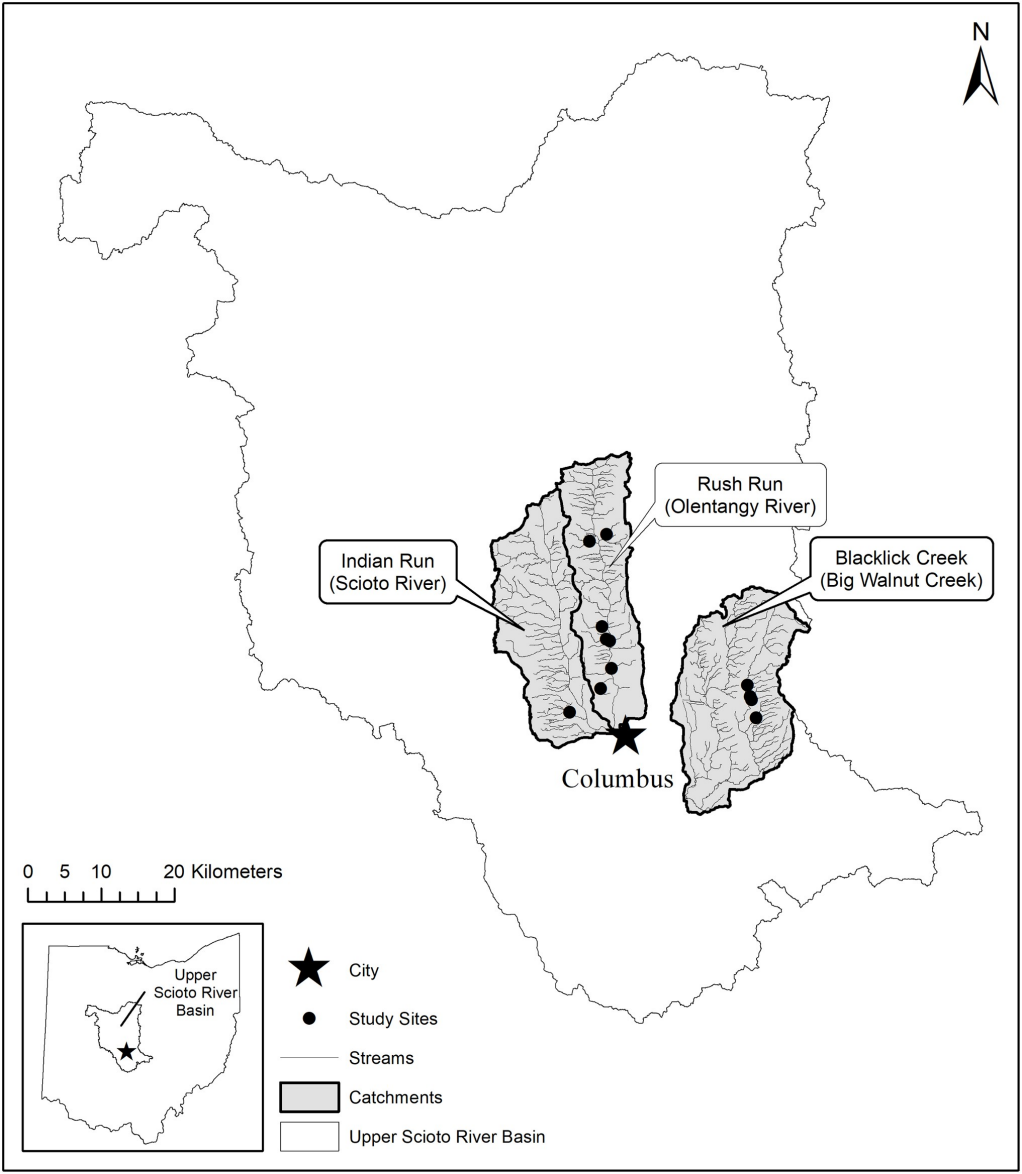
Map of study reach locations. Study reach locations (black dots) within major sub-catchments of the upper Scioto River basin. City center of Columbus, Ohio shown for reference.

Our study included twelve stream reaches in the Columbus Metropolitan Area (CMA), draining into the Olentangy or Scioto River basins with total catchment imperviousness ranging from 4.2 to 46.8% (Table 1). The CMA has a population of ~1.7 million people with an estimated 171,000 households and >27 km^2^ of net new commercial space projected to be added by 2040 [45]. The Scioto River is a 6^th^-order tributary of the Ohio River, and drains a 16,882-km^2^ catchment through central and southern Ohio (Fig 2). The Scioto River flows through Columbus in the upper part of the basin, which is characterized largely by row-crop agriculture north of the city and increasing suburban and urban land use in the CMA [45]. The Olentangy River (drainage area: 1,406 km^2^) also runs through Columbus, and empties into the Scioto River downtown.

**Table 1:** Land use/land cover information for all study reaches.

	Catchment				Riparian						
Site	% Developed	% Forest	% Agriculture	% Impervious	% Developed	% Forest	% Agriculture	% Develop pre-1990	% Develop 1991–2000	% Develop post-2001	Main Development Period
*Rush Run Catchment*											
Adena	97.3	2.7	0.0	37.5	82.6	17.4	0.0	100.0	0.0	0.0	Pre-1990
Big	21.1	20.0	53.3	5.9	17.0	79.1	3.5	51.0	2.0	47.0	Pre-1990
Kempton	79.7	9.9	7.7	20.7	44.7	41.4	10.9	87.0	10.0	3.0	Pre-1990
Leeds	12.1	2.8	63.9	46.8	11.9	24.4	36.7	24.0	18.0	58.0	Post-2001
Linworth	95.1	4.9	0.0	36.2	34.4	65.6	0.0	96.0	2.0	2.0	Pre-1990
Rush	94.5	5.4	0.0	4.9	70.5	28.1	0.0	92.0	6.0	2.0	Pre-1990
Waterman	82.1	17.9	0.0	32.5	52.8	47.2	0.0	–	–	–	–
*Blacklick Creek Catchment*											
Cole	23.7	42.5	32.8	6.1	21.3	53.5	24.1	58.0	15.0	27.0	Pre-1990
Dysart	55.9	15.0	24.8	16.9	52.8	29.1	11.9	17.0	19.0	63.0	Post-2001
Fieldstone	16.5	26.4	48.2	4.2	17.6	47.3	25.0	48.0	8.0	45.0	Pre-1990
Jefferson	43.3	5.5	36.1	17.2	48.1	24.8	25.4	5.0	0.0	95.0	Post-2001
*Indian Run Catchment*											
Slate	98.6	1.3	0.0	48.3	100.0	0.0	0.0	–	–	–	–

Percentage cover of urban/developed, forested, and agricultural land use, and impervious land cover within the riparian zone (i.e., 30 m buffer) and the catchment for each study reach listed by sub-catchment. Data derived from 2006 National Land Cover Database [46]. Percentage of currently developed land that was developed pre-1990, 1991–2000, and post-2001 derived from MacFarland [47], with main development period being when the majority of development activity occurred. “–” indicates no value available. Note: values not adding up to 100% indicate that other land use (grassland, wetland, open water, shrub/scrub) was present in catchment or riparian area. These categories are not listed due to low occurrence. “Develop” = Development.

The twelve study reaches were in small streams of the upper Scioto River basin, with seven streams entering directly into the Olentangy River, one into the Scioto River, and four into Blacklick Creek (Fig 2). Blacklick Creek drains 159 km^2^ of largely agricultural and suburban land before emptying into Big Walnut Creek, which flows into the Scioto River south of Columbus. Several study reaches flow through areas near downtown Columbus that have been developed for >100 years, others pass through suburban areas that were developed in the past 20 years, and others underwent an agricultural-to-suburban transition during the four-year study period.

Study reaches were all first- and second-order pool-riffle systems (drainage area: 0.4–7.4 km^2^) with natural substrate beds (i.e., not concrete) and were thus comparable in bed morphology and slope (Table 2). Development in study reach catchments was primarily residential, with minimal industrial land use. Variability in landscape settings resulted in a 74–94% range in canopy cover over the channel; canopy cover over the riparian zone (i.e., from stream bank to 30 m perpendicular away from the stream) ranged from 70–93% (Table 2). Reach lengths were selected to be 20-30x the stream’s estimated bankfull width [48, 49] for both geomorphic and ecologic sampling, and ranged from 130 to 390 m.

**Table 2:** Study reach characteristics.

Study reach	Drainage area (km^2^)	Stream order	Slope (%)	Stream canopy cover (%)	Riparian canopy cover (%)
*Rush Run Catchment*					
Adena	7	1	0.54	86.1	83.8
Big	6.5	1	0.89	83.1	89.3
Kempton	4.6	1	0.61	90.9	69.6
Leeds	4.9	1	1.07	84.9	89.3
Linworth	2.4	1	1.02	89.6	89.7
Rush	7.4	1	1.5	74.1	78.9
Waterman	0.4	1	1.3	93.9	74.6
*Blacklick Creek Catchment*					
Cole	3.2	2	0.91	87.7	74.4
Dysart	7.2	2	0.66	91	91.5
Fieldstone	4.9	2	1.23	90.8	91.7
Jefferson	2.2	1	1.5	91.5	92.9
*Indian Run Catchment*					
Slate	7.2	2	0.66	88.8	82.4

Drainage area, stream order, slope, canopy cover over the stream channel, and riparian canopy cover for Columbus, Ohio study reaches listed by catchment to which they contribute. Stream order was based on Strahler [50], using the 1:24,000-resolution National Hydrography Dataset [51].

Catchments draining to the terminal point of each reach were delineated using the StreamStats program for Ohio [52]. Drainage area, land use for both 2006 and 2011, and impervious surface area for 2011 were calculated using National Land Cover Databases [46, 53], using ArcGIS 10.3.1 (Esri, Redlands, California). Land use and impervious surface area were calculated for both the entire catchment and for the riparian zone (within a 30-m buffer surrounding the stream as shown in the National Hydrography Dataset [51]). Using these data, % forest, % developed, and % impervious surface were calculated for catchments and riparian zones. In a prior study [47], information from Franklin [54], Licking [55], and Delaware [56] Counties’ Auditor’s Offices were used to calculate the proportion of total development in each catchment that occurred prior to 1990 (prior to large-scale suburbanization), from 1991–2000, and after 2000 (during a boom in suburban housing construction) for 10 of the study reaches (Slate and Waterman were not included; Table 1). Using these available data, we then assigned each study reach into two categories (majority of development pre-1990 or majority of development post-2001) based on the time period during which the majority (i.e., > 50%) of total development occurred (no study reach experienced most of its catchment development in 1991–2000; Table 1).

### Field methods

Stream geomorphic features and fish assemblages were surveyed at all study reaches once annually from 2011 through 2013 during the summer or early fall (June-September). Four reaches that exhibited the largest observed shifts in hydrogeomorphic characteristics through 2013 were surveyed again in 2015. Both fish and geomorphic surveys were conducted in reaches standardized to the size of the stream, with each study reach being 20–30× the estimated bankfull width of the stream (see Study system and experimental design, above). Geomorphic surveys were conducted using a laser level (SPECTRA Self Leveling Laser, Trimble Construction Tools Division, Dayton, Ohio), a laser receiver affixed to a telescoping rod, 30-m measuring tapes, 254-cm rulers (for pebble counts), and a compass (see [57] for detailed methodology). Longitudinal surveys included measuring bed and water-surface elevation along the thalweg at each point of noticeable grade change, sinuosity (azimuth of stream at each point of bed elevation change along reach), and bankfull elevation at 4–10 points along the stream reach following Ward and Trimble [57]. Lateral surveys included three cross-sections measuring bed elevation across the estimated floodprone width of the stream, spaced evenly throughout the reach at riffles characteristic of the entire reach [57, 58]. A Wolman [59] pebble count of 100 bed particles was also conducted at each cross-section to characterize grainsize distribution (*n* = 300 particles per study reach). Survey data were entered into the Reference Reach Spreadsheet Version 4.3L ([60], further explained in [47]). This spreadsheet calculates multiple first-order (e.g., bankfull dimensions, slope, sinuosity) and second-order (e.g., velocity, stream power, discharge, threshold grain size) geomorphic and hydrologic variables (S1 Table) and has been used in other ecological studies (e.g., [61]).

For fish assemblages, each reach was isolated from upstream and downstream sections using block nets prior to fish collection. Fish were collected using a Smith-Root LR-24^®^ (Smith-Root, Inc., Vancouver, Washington) backpack electrofisher by performing three separate passes at each reach; this depletion level acceptably represents community-level fish data in small streams of the region (e.g., [62]). Individual fish were counted, identified to species, weighed, and measured (total length; weight and length data not used here). Data were used to calculate fish density (S2 Table); species richness (*S*; S2 Table); the Shannon-Wiener diversity index (*H’*; [63, 64]; S2 Table); and relative abundances (i.e., proportions) of trophic generalists, herbivores, pollution tolerant/intolerant species, and the number of darter species as classified by the Ohio Environmental Protection Agency [65].

Canopy cover over both banks of the stream, as well as over the stream, was measured using a densiometer following methods outlined in Lemmon ([66]; utilized in streams in [67–69]). Percentage canopy coverage was calculated every 25 m over the entire reach, or up to 200 m centered in the middle of longer reaches. At each 25-m measurement point, canopy coverage was measured facing each of the four cardinal directions. Mean canopy cover on both banks and along the thalweg could then be calculated for each study reach. Riparian canopy cover was also measured at four transects along each study reach following methodology outlined in Lemmon [66]. Transects alternated between right and left banks (two transects on right bank, two transects on left for each site). At each transect, measurements were made at 10, 20, and 30 m from stream bank into the riparian zone, and similar to stream canopy cover measurements, riparian canopy cover was measured facing each of the cardinal directions at each measurement point.

Chemical water-quality and nutrient samples were collected in 2014, 2016, 2017, and 2018 at each study reach at 5–7 locations. Sub-samples from each reach were composited by reach and were refrigerated before submission to The Ohio State University’s Service Testing and Research (STAR) Laboratory for analysis of total nitrogen (N; mg L^-1^), total phosphorus (P; mg L^-1^), total dissolved solids (TDS; mg L^-1^), and total Hg (ppt) within 24 h of collection. Samples were analyzed for Hg using cold vapor atomic fluorescence. Instrument calibration (CETAC M8000 mercury analyzer, CETAC Technologies, Omaha, Nebraska) was performed with NIST-traceable 100 mg L^-1^ mercury standard (SPEX CertiPrep, Metuchen, New Jersey). Samples were digested using a 1:1 mixture of trace metal grade perchloric acid and nitric acid. Lab reagent blanks (manufactured in-house; 3% hydrochloric acid added to nanopure water) and NIST Standard Reference Materials were analyzed for quality assurance during invertebrate sample and sediment sample sequences (1547 peach leaves and 2709a San Joaquin soil, respectively). Total N and P were analyzed using flow injection analysis (Latchat Quick Chem 8500 Flow Injection Analyzer, Hach Company, Loveland, Colorado), while TDS was measured using a drying oven and scale. Total N and total P were highly correlated (*p* < 0.0001, *R*^*2*^ = 0.99).

### Statistical methods

Based on preliminary work at a subset of the current study reaches (Rieck and Sullivan, unpublished data), ten predictor variables related to stream hydrogeomorphic features were included in the analyses: discharge (m^3^ s^-1^); shear stress (kPa); bankfull width (m); width:depth, entrenchment, and incision ratios; sinuosity; D_50_ (mm); slope (%); and width-of-floodprone area (m). Principal component analysis was used to investigate any potential groupings of sites based on hydrogeomorphic characteristics, with no strong patterns of groups emerging (S1 Fig). Fish response variables included species richness (*S*), diversity (*H’*), % trophic generalists, % herbivores, % highly tolerant, no. darter species, and density (no. m^-3^). Differences in fish variables from 2011–2013 were also calculated (e.g., Δ value = 2013 value– 2011 value) to be used as response variables in subsequent models.

To address the prediction that fish assemblages would reflect urban-induced shifts in hydrogeomorphic characteristics, relationships between hydrogeomorphic predictors and fish assemblage composition were initially investigated using canonical correspondence analysis (CCA) for 2011, 2012, and 2013 individually (package vegan [70] in R [71]). CCA is a common ordination technique to explore associations between environmental variability and biotic communities (e.g., [72]). Hydrogeomorphic predictor variables included in CCA were reduced to keep variance inflation factors (VIF; used to evaluate multicollinearity) < 10 [73] by alternately eliminating variables to best retain variation but reduce VIF, resulting in a set of seven hydrogeomorphic predictors: width-of-floodprone area (m), sinuosity, incision ratio, entrenchment ratio, discharge (m^3^ s^-1^), D_50_ (mm), and slope (%).

To address our second set of predictions (regarding specific shifts in fish assemblage metrics in response to types and magnitudes of hydrogeomorphic adjustment), we used the reduced set of seven hydrogeomorphic predictors to focus on three types of hydrogeomorphic adjustment: (1) floodplain connectivity (width-of-floodprone area, sinuosity, incision ratio, width:depth ratio); (2) hydrologic variability (discharge); and (3) bed characteristics (D_50_, slope). We then used generalized linear models (GZLM; glm function) to assess the impact of these three hydrogeomorphic features, along with year and riparian canopy cover, on Δ values of fish assemblage characteristics. The best distributions for all response variables were obtained using fitdistrplus [74], with all response variables best fit by a binomial logit distribution. Relative support for each of the respective eight models ([1] year only; [2] riparian canopy cover and year; [3] floodplain connectivity and year; [4] hydrologic variability and year; [5] bed characteristics and year; [6] floodplain connectivity, riparian canopy, and year; [7] hydrologic variability, riparian canopy, and year; and [8] bed characteristics, riparian canopy, and year) were then compared using AICc, with ΔAICc ≤ 2 considered notable support [75, 76]. 2015 data were plotted and visually assessed along with data from other years for potential patterns. Trends in mean values for both predictor and response variables were assessed using ANOVA (aov function) followed by linear contrasts in R for 2011, 2012, and 2013. All analyses for predictions 1 and 2 were run in R [71].

Our third set of predictions were exploratory in nature and meant to take an initial step at identifying potential broad-scale mechanisms driving observed changes in hydrogeomorphic features and fish assemblages. To do this, we used simple linear regression with impervious surface and % development pre-1990 as predictors of a subset of hydrogeomorphic (width-of-floodprone area, incision ratio, and D_50_) and fish (*H*’ [as a general characterization of the assemblage], % generalists, % specialists) variables. These response variables were primarily selected based on results of GZLMs run for the second prediction (see above). To preliminary assess the potential influence of water chemistry and nutrients on fish assemblages, we performed principal component analysis (PCA) on all water-chemistry variables from 2014 to reduce dimensionality among these variables. PCA axes with eigenvalues >1 were retained for use in subsequent linear regression models [77, 78] with *H*’, %generalists, % specialists as response variables.

In addition, we visually assessed trends in chemical water-quality and nutrient variables using samples collected in 2014, 2016, 2017, and 2018 to provide context relative to general water-chemistry variability in the study system (S2 Fig). All statistical analyses for the third set of predictions were performed using JMP 12 (SAS Institute, Cary, North Carolina). For all tests, significance was determined at α = 0.05, with α = 0.10 considered evidence of a trend.

## Results and discussion

### Results

#### Hydrogeomorphic characteristics, chemical water quality, and nutrients

**Fig 3:**
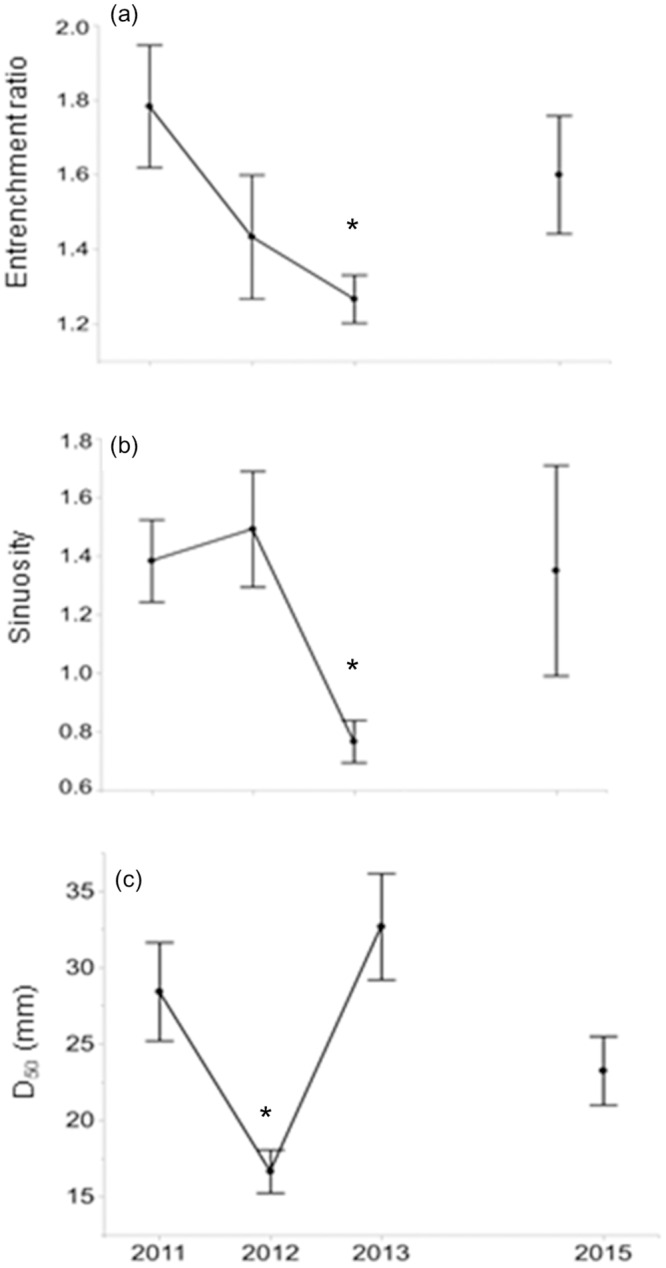
Line graphs of hydrogeomorphic predictors by year. Average values +/- 1 SE for three hydrogeomorphic variables showing significant differences between years examined at the 12 study sites from 2011–2013, and in 2015 (four sites only): (a) entrenchment ratio, (b) sinuosity, and (c) D_50_ (mm). Asterisk indicates year that was significantly different.

Whereas some hydrogeomorphic variables showed considerable variation across the 3–5 years of the study, others were more consistent (Table 3). For instance, width-of-floodprone area ($\bar{x}=12.4\;\text{m}$, SE = 1.9) and discharge ($\bar{x}=3.44{\;\text{m}}^{3}{\text{s}}^{-1}$, SE = 0.51) showed high variability, while D_50_ ($\bar{x}=25.7\;\text{mm}$, SE = 1.8) and slope ($\bar{x}=1.00\%$, SE = 0.06) exhibited lower variability across our study reaches (Table 3). Sinuosity showed a wide range across sites, from 0.90 at Slate to 2.25 at Big, and width:depth ratios ranged from 13.8 at Cole to 65.6 at Rush (S3 Table). We also observed some overall directional changes in hydrogeomorphic characteristics (Fig 3, S3 Fig, S4 Table): entrenchment ratio (Fig 3a) decreased from 2011–2013, while sinuosity (Fig 3b) decreased only in 2013, and D_50_ (Fig 3c) decreased in 2012 before rebounding in 2013.

**Table 3:** Descriptive statistics for predictor and response variables.

Variable	Mean	SE	Min	Median	Max
*Predictors–Hydrogeomorphic characteristics*					
Width-of-floodprone area (m)	12.4	1.90	4.6	9.8	83.2
Discharge (m^3^ s^-1^)	3.44	0.51	0.31	2.57	17.93
Shear stress (kPa)	0.029	0.00	0.005	0.028	0.068
Bankfull width (m)	8.3	1.00	3.0	7.1	45.6
Width:depth ratio	28.3	4.30	11.8	22.4	186.4
Entrenchment ratio	1.5	0.08	0.9	1.4	2.9
Incision ratio	1.8	0.08	1.1	1.7	3.2
Sinuosity	1.2	0.09	0.5	1.1	2.9
D_50_ (mm)	25.7	1.80	10.0	23.0	51.0
Slope (%)	1.00	0.06	0.15	0.95	2.00
*Responses–Fish*					
Species richness (*S*)	7.1	0.70	0.0	7.5	16.0
Diversity (*H’*)	0.990	0.09	0.000	1.027	1.955
% Trophic generalists	60.5	1.80	0	60.78	100
% Herbivore	9.7	0.28	0.0	5.4	39.8
% Highly tolerant	70.9	1.90	0	75.9	100.0
No. darter species	1.2	0.51	0.0	0.0	5.0
Density (no. m^-3^)	1.0	0.06	0	0.7	7.5

Descriptive statistics for predictor and response variables included in the analyses, including mean, standard error (SE), minima (min), median, and maximum (max) for each variable.

Some study reaches (e.g., Slate and Rush) showed large adjustments during the study period, but in opposite directions: e.g., Slate experienced an increase in width-of-floodprone area and decreases in discharge, shear stress, and slope, while Rush experienced a decrease in width-of-floodprone area and increases in discharge, shear stress, and slope. Other study reaches (e.g., Waterman) showed little change in multiple characteristics (but note that slope and incision ratio decreased; S5 Table).

Across the twelve study reaches, means for N, P, Hg, and TDS were 1.30 mg L^-1^, 0.44 mg L^-1^, 0.002 ppt, and 298.8 mg L^-1^ for 2014 water samples, respectively (S6 Table). Interannual variation in chemical water-quality and nutrient variables at each study reach was relatively low in general, suggesting that 2014 values were a reasonable approximation of water quality across the reaches during the period of the study (S2 Fig).

#### Fish-assemblage characteristics

We captured a mean of 1.03 fish (no. m^-3^) across all sites over the course of study, ranging from 0 (Kempton 2011 and Waterman 2011–2012) to 7.48 no. m^-3^ (Rush 2015; Table 3 and S7 Table). Species richness was relatively low, with a mean of 7.1 species, ranging from 0–16 species per study reach (Table 3). Highly tolerant species were most commonly Creek Chub (*Semolitus atromaculatus*), Green Sunfish (*Lepomis cyanellus*), or White Sucker (*Catostomus commersonii*); these common species represented 70.9% of the assemblage on average across all reaches (Table 3).

**Fig 4:**
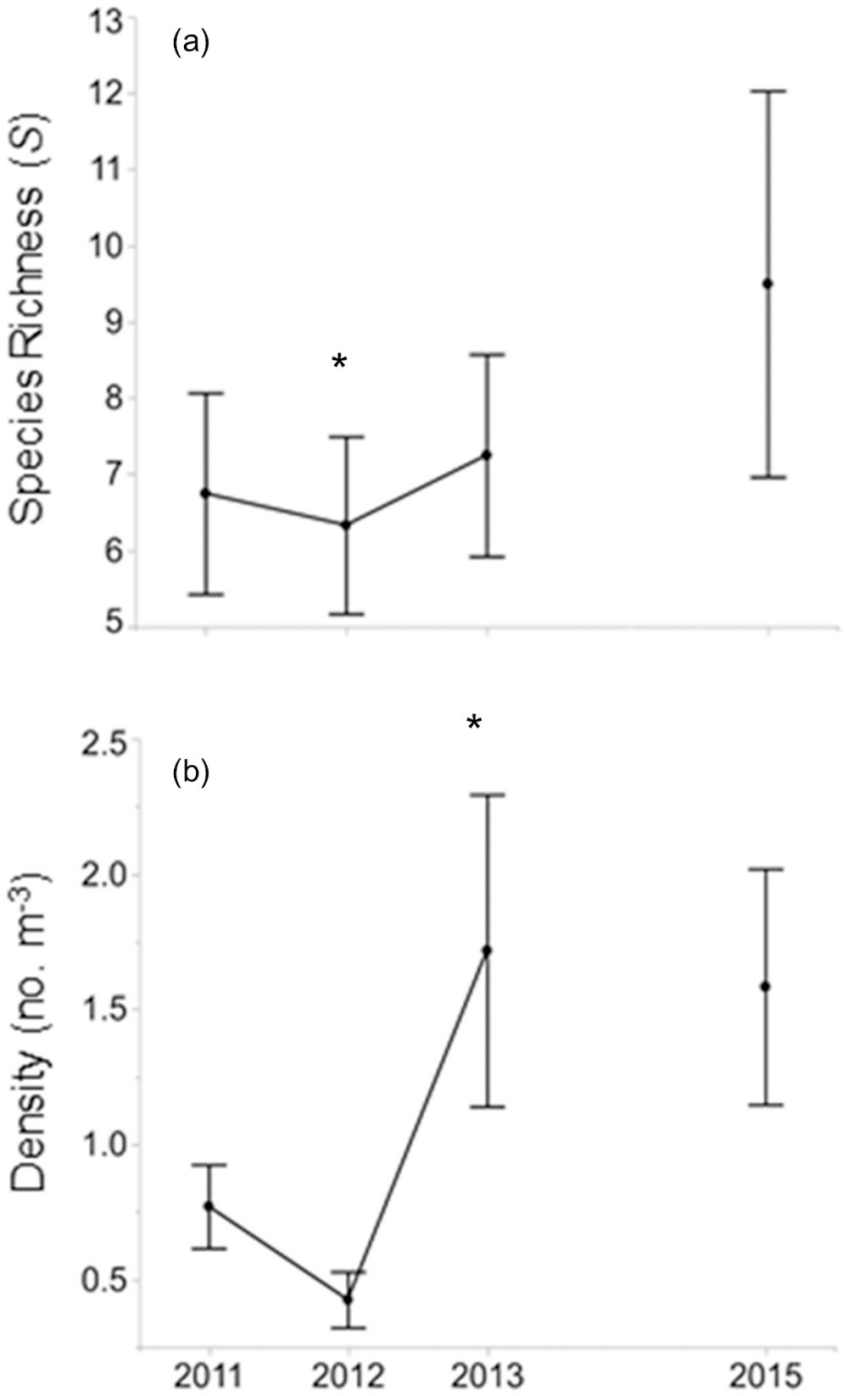
Line graphs of fish assemblage variables by year. Average values +/- 1 SE for the two fish assemblage variables showing significant differences between years at the 12 study reaches from 2011–2013, and in 2015 (four sites only: (a) species richness (*S*) (b) density (no. m^-3^). Asterisk indicates year that was significantly different.

Similar to hydrogeomorphic variables, some fish-assemblage characteristics showed directional patterns over the study years (e.g., species richness [Fig 4a] and density [Fig 4b]), though most showed high variability and no directional pattern (S4 Fig and S4 Table). Change values (i.e., Δ from 2013–2011) displayed notable trends. Species richness showed an average gain of 0.5 species over the study period, but with a broad range (a loss of 7 species at Dysart, a gain of 9 species at Rush; S5 and S8 Tables). This wide range despite little change in mean values was further illustrated by diversity (*H’*; Table 3 and S8 Table).

#### Species-specific responses to hydrogeomorphic features

**Fig 5:**
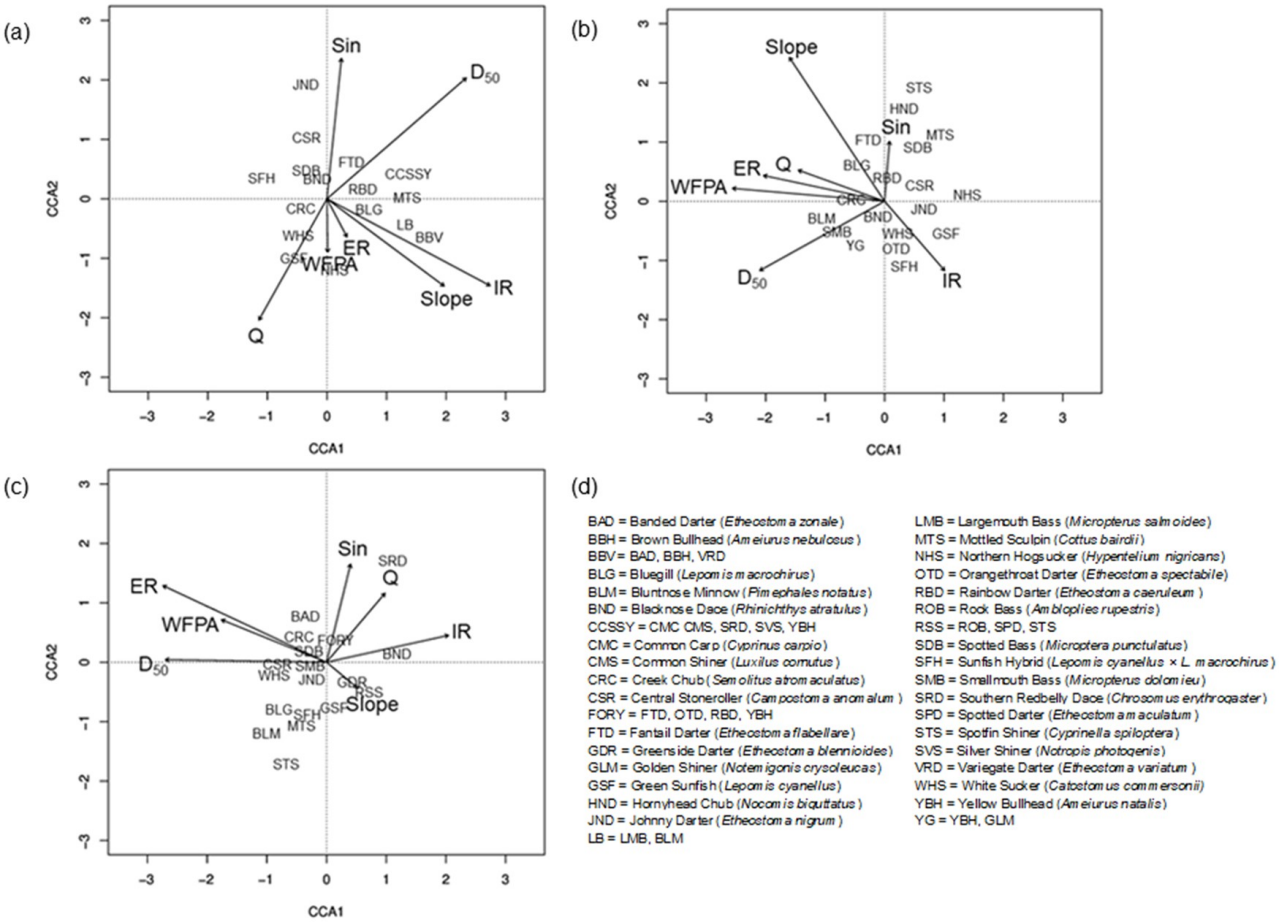
Ordination biplots from canonical correspondence analysis by year. Ordination biplots based on canonical correspondence analysis of seven hydrogeomorphic variables that minimize variance inflation factors and the relative abundance of fish species in (a) 2011, (b) 2012, and (c) 2013. Panel (d) provides key to species abbreviations.

Canonical correspondence analysis (CCA) provided valuable information regarding shifts in relative abundances of fish species in relation to hydrogeomorphic features in each year (prediction 1). The primary axis of the 2011 CCA explained 32.9% of the variance in hydrogeomorphic characteristics, while the secondary explained 16.5%. Notable fish-hydrogeomorphic relationships included a positive relationship between Johnny Darter (*Etheostoma nigrum*) relative abundance and sinuosity as well as between Banded Darter (*Etheostoma zonale*), Brown Bullhead (*Ameiurus nebulosus*), Variegate Darter (*Etheostoma variatum*), Largemouth Bass (*Micropterus salmoides*), and Bluntnose Minnow (*Pimephales notatus*) relative abundances and slope and incision ratio (Fig 5a). The latter group contains species known to be insectivores, with some species (e.g., Largemouth Bass) transitioning to piscivores at larger sizes [79].

The 2012 CCA had a primary axis explaining 27.4%, and a secondary axis explaining 13.4%, of the variation in hydrogeomorphic characteristics. In contrast to the 2011 CCA, in 2012 Johnny Darter relative abundances were negatively associated with discharge, entrenchment ratio, and width-of-floodprone area, while relative abundances of White Sucker, Orangethroat Darter (*Etheostoma spectabile*), Sunfish Hybrids (*Lepomis cyanellus* × *L*. *macrochirus*), and Green Sunfish were positively associated with incision ratio (Fig 5b). Within the latter group, all species are insectivores with the exception of White Suckers, and all are pollution-tolerant with the exception of Orangethroat Darter [65, 79]. CCA for 2013 produced a primary axis explaining 34.0% of the variance in hydrogeomorphic characteristics and a secondary axis explaining 21.2%.

The 2013 CCA was notably different from those in 2011 and 2012 in that Johnny Darter relative abundances showed no notable associations, while relative abundances of Mottled Sculpin, Bluegill (*Lepomis macorchirus*), Bluntnose Minnow, Spotfin Shiner (*Cyprinella spiloptera*), and Sunfish Hybrids (all insectivores [65, 79]) were negatively associated with sinuosity, discharge, and incision ratio (Fig 5c). Relative abundances of Southern Redbelly Dace (*Chrosomus erythrogaster*) were closely positively related to discharge (Fig 5c).

#### Fish assemblage responses to hydrogeomorphic features

Hydrogeomorphic variables emerged as salient, although not always significant, predictors of fish assemblage metrics in multiple instances (prediction 2; Table 4 and S9 Table). Model selection indicated that at least two hydrogeomorphic models were well-supported for all fish assemblage characteristics (Table 4). “Hydrologic variability + Year” as well as “Bed Characteristics + Year” both showed support with Δ species richness (ΔAICc = 0.00 and 0.74, respectively) and diversity (*H’*; ΔAICc = 1.93 and 0.00, respectively). Δ diversity (*H’*) also showed a positive trend with D_50_ in the “Bed Characteristics + Year + Riparian Canopy” model (ΔAICc = 1.18). “Bed Characteristics + Year” was best supported for Δ % Generalists (ΔAICc = 0.00), displaying a negative trend with D_50_. Δ % Herbivores was best modeled by “Year-only” (ΔAICc = 0.00), although the “Hydrologic Variability + Year” model was also supported (ΔAICc = 0.80). Δ % Tolerant was best modeled by the “Bed Characteristics + Year + Riparian Canopy” model (ΔAICc = 0.00), with a significant negative relationship with riparian canopy cover and a negative trend with D_50_, while the “Riparian Canopy + Year” model was also supported and showed the same negative relationship with riparian canopy cover (ΔAICc = 1.28, Table 4). The “Hydrologic Variability + Year” model was supported for both Δ no. of darter species (ΔAICc = 0.29) and Δ density (ΔAICc = 1.51). The “Riparian Canopy + Year” model was also supported for Δ density (ΔAICc = 0.34), but no variable (i.e., neither % riparian canopy cover nor year) was significant within that model (Table 4).

**Table 4:** Linear models for influences of hydrogeomorphic features on fish assemblage characteristics that showed moderate support (ΔAICc ≤ 4). ΔAICc = AICc (lowest)—AICc (model x).

Response	Model	Predictor	Coef	*Z*	*p*	ΔAICc
*Δ Species Richness*	*Year-Only*	2012	< 0.01	0.00	1.000	0.04
		2013	< 0.01	0.00	1.000	
	*Hydrologic Variability + Year*	Discharge (m^3^ s^-1^)	0.16	0.98	0.327	0.00
		2012	-0.16	-0.19	0.850	
		2013	0.20	0.23	0.816	
	*Bed Characteristics + Year*	D_50_ (mm)	0.02	0.60	0.550	0.74
		Slope (%)	0.91	0.91	0.365	
		2012	0.36	0.38	0.703	
		2013	-0.01	-0.01	0.995	
*Δ Diversity (H’)*	*Year-Only*	2012	< 0.01	0.00	1.000	0.32
		2013	< 0.01	0.00	1.000	
	*Riparian Canopy + Year*	Riparian Canopy Cover (%)	-0.03	-0.57	0.568	2.07
		2012	< 0.01	0.00	1.000	
		2013	< 0.01	0.00	1.000	
	*Hydrologic Variability + Year*	Discharge (m^3^ s^-1^)	0.12	0.77	0.444	1.93
		2012	-0.12	-0.14	0.886	
		2013	0.15	0.18	0.857	
	*Bed Characteristics + Year*	D_50_ (mm)	0.07	1.61	0.107	0.00
		Slope (%)	0.63	0.61	0.540	
		2012	0.86	0.86	0.389	
		2013	-0.24	-0.26	0.797	
	*Hydrologic Variability + Year + Riparian Canopy*	Discharge (m^3^ s^-1^)	0.15	0.90	0.369	3.37
		2012	-0.15	-0.17	0.454	
		2013	-0.19	0.21	0.864	
		Riparian Canopy Cover (%)	-0.03	-0.75	0.830	
	*Bed Characteristics + Year + Riparian Canopy*	D_50_ (mm)	0.07	1.70	0.090	1.18
		Slope (%)	0.80	0.76	0.447	
		2012	0.92	0.92	0.358	
		2013	-0.24	-0.26	0.794	
		Riparian Canopy Cover (%)	-0.04	-0.92	0.355	
*Δ % Generalists*	*Year-Only*	2012	< 0.01	0.00	1.000	2.69
		2013	< 0.01	0.00	1.000	
	*Riparian Canopy + Year*	Riparian Canopy Cover (%)	-0.04	-0.73	0.463	3.32
		2012	< 0.01	0.00	1.000	
		2013	< 0.01	0.00	1.000	
	*Floodplain Connectivity + Year*	Width-of-floodprone area (m)	-0.13	-0.69	0.492	2.12
		Sinuosity	0.40	0.36	0.722	
		Incision Ratio	2.13	1.92	0.055	
		Width:depth Ratio	0.18	0.12	0.902	
		2012	0.11	0.08	0.937	
		2013	-0.60	-0.42	0.678	
	*Hydrologic Variability + Year*	Discharge (m^3^ s^-1^)	-0.20	-0.81	0.416	3.97
		2012	0.15	0.14	0.885	
		2013	-0.22	-0.21	0.837	
	*Bed Characteristics + Year*	D_50_ (mm)	-0.11	-1.86	0.063	0.00
		Slope (%)	1.69	1.18	0.237	
		2012	-0.91	-0.77	0.443	
		2013	0.62	0.53	0.597	
	*Bed Characteristics + Year + Riparian Canopy*	D_50_ (mm)	-0.11	-1.79	0.073	1.40
		Slope (%)	1.81	1.26	0.207	
		2012	-0.80	-0.67	0.504	
		2013	0.68	0.57	0.570	
		Riparian Canopy Cover (%)	-0.04	-0.62	0.532	
*Δ % Herbivores*	*Year-Only*	2012	< 0.01	0.00	1.000	0.00
		2013	< 0.01	0.00	1.000	
	*Riparian Canopy + Year*	Riparian Canopy Cover (%)	0.02	0.51	0.612	3.26
		2012	< 0.01	0.00	1.000	
		2013	< 0.01	0.00	1.000	
	*Hydrologic Variability + Year*	Discharge (m^3^ s^-1^)	-0.15	-0.89	0.371	0.80
		2012	0.16	0.17	0.864	
		2013	-0.20	-0.21	0.833	
	*Bed Characteristics + Year*	D_50_ (mm)	0.00	-0.09	0.925	2.92
		Slope (%)	1.22	1.10	0.270	
		2012	0.12	0.11	0.910	
		2013	0.14	0.15	0.880	
*Δ % Tolerant*	*Riparian Canopy + Year*	Riparian Canopy Cover (%)	-0.17	-2.67	0.008	1.28
		2012	< 0.01	0.00	1.000	
		2013	< 0.01	0.00	1.000	
	*Hydrologic Variability + Year + Riparian Canopy*	Discharge (m^3^ s^-1^)	-0.20	-0.75	0.455	3.26
		2012	0.09	0.08	0.934	
		2013	-0.17	-0.15	0.878	
		Riparian Canopy Cover (%)	-0.15	-2.38	0.018	
	*Bed Characteristics + Year + Riparian Canopy*	D_50_ (mm)	-0.12	-1.93	0.054	0.00
		Slope (%)	0.50	0.35	0.729	
		2012	-0.99	-0.75	0.453	
		2013	0.88	0.64	0.524	
		Riparian Canopy Cover (%)	-0.18	-2.50	0.013	
*Δ No*. *darter species*	*Year-Only*	2012	< 0.01	0.00	1.000	0.00
		2013	< 0.01	0.00	1.000	
	*Riparian Canopy + Year*	Riparian Canopy Cover (%)	-0.03	-0.60	0.547	3.28
		2012	< 0.01	0.00	1.000	
		2013	< 0.01	0.00	1.000	
	*Hydrologic Variability + Year*	Discharge (m^3^ s^-1^)	0.16	0.97	0.331	0.29
		2012	-0.16	-0.19	0.852	
		2013	0.20	0.23	0.817	
	*Bed Characteristics + Year*	D_50_ (mm)	0.02	0.56	0.577	3.59
		Slope (%)	1.07	1.06	0.290	
		2012	0.36	0.38	0.703	
		2013	0.02	0.02	0.983	
	*Hydrologic Variability + Year + Riparian Canopy*	Discharge (m^3^ s^-1^)	0.20	1.11	0.267	3.64
		2012	-0.20	-0.23	0.819	
		2013	0.23	0.27	0.787	
		Riparian Canopy Cover (%)	-0.04	-0.83	0.404	
*Δ Density (no*. *m*^*-3*^*)*	*Year-Only*	2012	< 0.01	0.00	1.000	0.00
		2013	< 0.01	0.00	1.000	
	*Riparian Canopy + Year*	Riparian Canopy Cover (%)	-0.05	-0.87	0.385	0.34
		2012	< 0.01	0.00	1.000	
		2013	< 0.01	0.00	1.000	
	*Floodplain Connectivity + Year*	Width-of-floodprone area (m)	-0.06	-0.41	0.680	2.45
		Sinuosity	-0.67	-0.66	0.510	
		Incision Ratio	-1.90	-1.49	0.136	
		Width:depth Ratio	0.95	0.99	0.324	
		2012	0.05	0.05	0.962	
		2013	0.42	0.30	0.765	
	*Hydrologic Variability + Year*	Discharge (m^3^ s^-1^)	-0.17	-0.73	0.468	1.51
		2012	0.13	0.13	0.897	
		2013	-0.19	-0.18	0.857	
	*Bed Characteristics + Year*	D_50_ (mm)	0.07	1.39	0.165	2.30
		Slope (%)	-1.11	-0.90	0.370	
		2012	0.75	0.60	0.551	
		2013	-0.39	-0.36	0.717	
	*Hydrologic Variability + Year + Riparian Canopy*	Discharge (m^3^ s^-1^)	-0.13	-0.55	0.581	2.67
		2012	0.09	0.09	0.932	
		2013	-0.14	-0.13	0.894	
		Riparian Canopy Cover (%)	-0.04	-0.70	0.486	
	*Bed Characteristics + Year + Riparian Canopy*	D_50_ (mm)	0.07	1.47	0.143	2.57
		Slope (%)	-0.93	-0.76	0.450	
		2012	0.76	0.61	0.540	
		2013	-0.47	-0.42	0.672	
		Riparian Canopy Cover (%)	-0.05	-0.92	0.359	

Model selection results for generalized linear models with ΔAICc ≤ 4. Gray shading indicates best model (lowest AICc, or, if not “year-only” model, ΔAICc ≤ 2) as well as individual hydrogeomorphic predictors showing significant or trending relationships with fish assemblage characteristics.

#### Mechanisms driving hydrogeomorphic and fish assemblage changes

**Fig 6:**
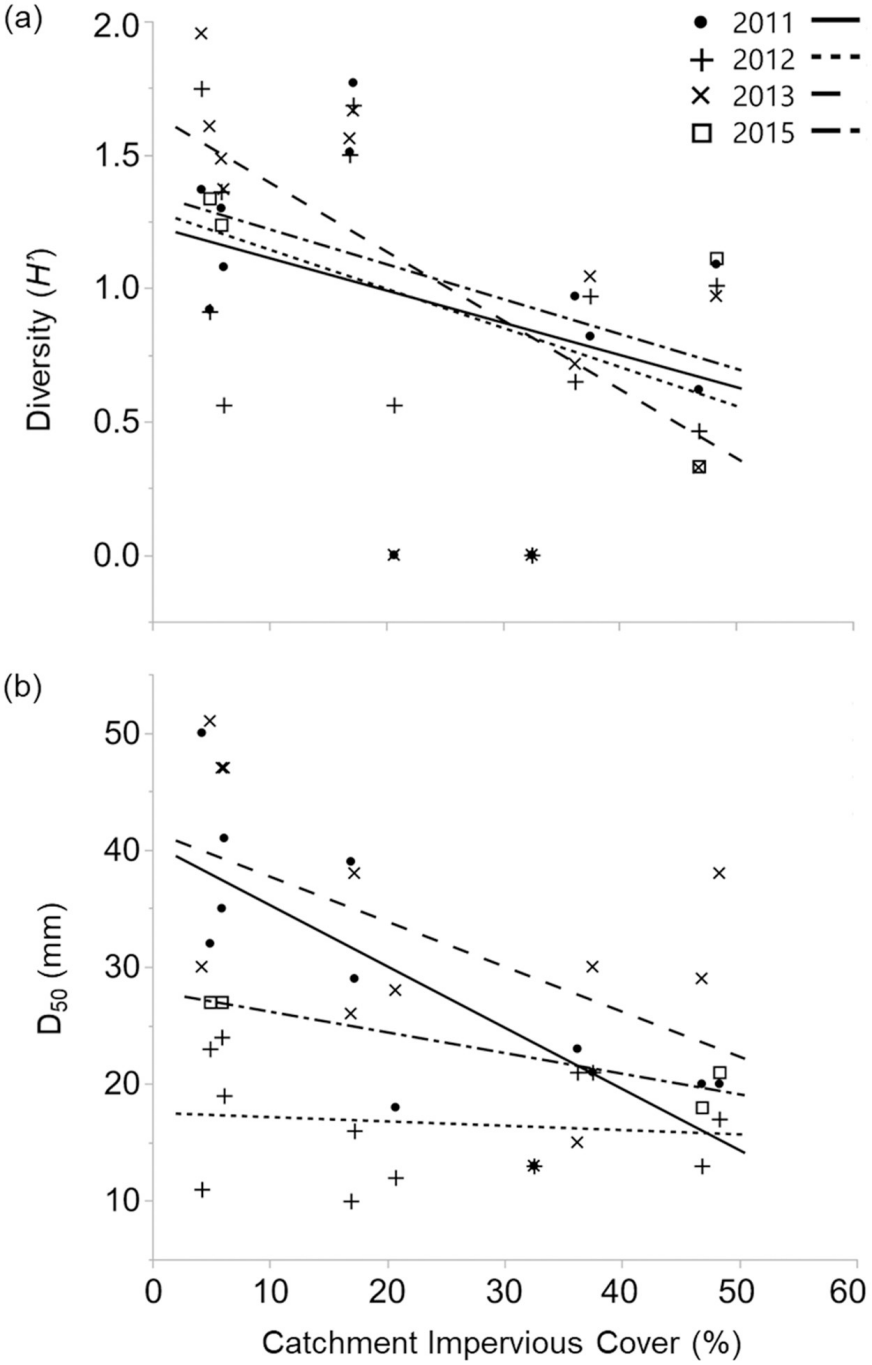
Linear relationships between % catchment imperviousness and Δ fish assemblage and hydrogeomorphic variables. Linear relationships by year between % catchment imperviousness and (a) diversity (*H’*; 2011: *R*^*2*^ = 0.14, *F* = 1.57, *p* = 0.239; 2012: *R*^*2*^ = 0.20, *F* = 2.56, *p* = 0.141; 2013: *R*^*2*^ = 0.41, *F* = 6.96, *p* = 0.025; 2015: *R*^*2*^ = 0.48, *F* = 1.88, *p* = 0.304) and (b) D_50_ (2011: *R*^*2*^ = 0.61, *F* = 15.33, *p* = 0.003; 2012: *R*^*2*^ = 0.02, *F* = 0.16, *p* = 0.699; 2013: *R*^*2*^ = 0.28, *F* = 3.87, *p* = 0.077; 2015: *R*^*2*^ = 0.91, *F* = 20.72, *p* = 0.045).

The first principal component (PC) axis represented 60.1% of the variance in the water-quality data set and was primarily influenced by total N and P (loadings were 0.597 for both; S10 Table). However, we found no association between this PC and any fish assemblage characteristics (Table 5), suggesting that water quality was not a key mechanism (prediction 3) driving differences in measured fish assemblage metrics in our study. In contrast, Δ diversity (*R*^*2*^ = 0.55, *F* = 42.04, *p* < 0.001) was negatively associated with percentage catchment imperviousness, but this relationship varied by year (Table 5; Fig 6a). % development pre-1990 and % riparian canopy cover were not related to width-of-floodprone area, incision ratio, or D_50_ (i.e., the three hydrogeomorphic variables that emerged as salient in fish-hydrogeomorphic linear models) (Table 4). However, % catchment impervious cover was negatively associated with D_50_ (Table 4; Fig 6b).

**Table 5:** Simple linear regression results for fish assemblage and hydrogeomorphic Δ variables with land use and water-chemistry variables.

	% Develop Pre-1990				% Imperviousness				Water Quality PC			
	Direction	*R*^*2*^	*F*	*P*	Direction	*R*^*2*^	*F*	*p*	Direction	*R*^*2*^	*F*	*p*
Δ Diversity (*H’*)	+	0.08	0.73	0.419	-	0.55	12.36	0.006	+	0.04	0.46	0.512
Δ % Generalists	-	0.26	2.78	0.134	+	0.09	0.97	0.347	+	0.11	1.17	0.304
Δ % Tolerant	+	0.03	0.27	0.615	+	0.02	0.20	0.664	-	< 0.01	< 0.01	0.983
Δ Width-of-floodprone area (m)	-	0.04	0.33	0.579	+	0.12	1.35	0.272	-	-	-	-
Δ Incision ratio	+	0.04	0.36	0.566	+	< 0.01	0.02	0.904	-	-	-	-
Δ D_50_ (mm)	+	0.05	0.42	0.533	+	0.04	0.37	0.556	-	-	-	-

Simple linear regression results for Δ diversity (*H’*), Δ % generalists, and Δ % tolerant with % catchment development pre-1990, % catchment imperviousness, and the first water-chemistry principal component and for Δ width-of-floodprone area, Δ incision ratio, and Δ D_50_ with % catchment development pre-1990 and % catchment imperviousness. Direction indicates nature of relationship. Gray shading indicates significant relationship.

### Discussion

From our Columbus, Ohio streams, we found multiple lines of evidence that supported our overall hypothesis that fish assemblage characteristics would reflect changes in stream geomorphic features, though evidence was lacking to support many of our specific predictions. We found multiple associations between individual species and hydrogeomorphic characteristics, but the nature of these relationships varied over time, highlighting the nuanced habitat-species relationships mediated by fluvial geomorphology. At the community level, three main categories of hydrogeomorphic features were evaluated for their influence on fish assemblages: (1) floodplain connectivity (e.g., width-of-floodprone area, incision ratio, sinuosity), (2) hydrologic variability (e.g., discharge), and (3) bed characteristics (e.g., D_50_, slope). Linear models indicated that median sediment size and incision ratio were related to both % generalists and % tolerant species, presenting interesting relationships. However, these results were unexpectedly the only significant relationships found within best-supported models, leaving predictions regarding fish diversity, density, and the abundance of invertivores largely unsupported. Floodplain connectivity and landscape features (i.e., land use/land cover and development patterns) can be important drivers of in-stream hydrogeomorphic conditions that influence fish assemblages more directly [80]. For example, we found that % impervious surface in the catchment was related to both sediment grain size distribution (D_50_) and fish diversity (*H*’), suggesting potential mechanisms through which land use cascades through hydrogeomorphic and habitat change to impact fish assemblages that should be further explored. We did not observe that chemical water quality exerted a strong influence on fish assemblages. However, increased temporal sampling of water chemistry would be necessary to make stronger inferences on the relative impacts of hydrogeomorphic characteristics and chemical water quality on urban fish assemblages.

#### Urban stream geomorphology

Urban stream channels are often enlarged (i.e., increased width:depth ratio) and entrenched (i.e., increased entrenchment ratio) relative to unimpacted channels owing to characteristic urban flow regimes, characterized by higher bankfull discharge and shear stress and lower baseflow discharge (reviewed in [81, 82]). Findings from a variety of urban systems support this generalization (e.g., [21, 83, 84]), yet channel enlargement is not a ubiquitous response to urbanization [85, 86]. Although we observed some channel enlargement responses, we also observed wide spatiotemporal variability in hydrogeomorphic processes impacting the measured characteristics (Fig 3). For instance, while mean width:depth ratios and bankfull width increased from 2011–2015, incision ratios and D_50_ showed no consistent pattern across study years (Fig 3), suggesting that some changes (e.g., increasing width over time) may be consistent in urban watersheds of the study, while others are more sensitive to site-specific characteristics.

Multiple local-scale landscape factors, including local geology [87, 88], sediment regimes [23, 89, 90], and riparian vegetation [91, 92] may influence the type and rate of channel response to development. Acknowledging these and other variables, Booth and Fischenich [93] provided an updated channel evolution model (CEM) specifically for urban streams, considering channel type (alluvial vs. non-alluvial; single-thread vs. braided), valley morphology, channel confinement (anthropogenic or natural), and anthropogenic activities impacting fluvial geomorphic responses. Many of the variables (e.g., anthropogenic in-stream structures, valley morphology) considered in the Booth and Fischenich [93] urban CEM likely influenced the variability we observed in hydrogeomorphic alterations. For instance, Rush was heavily influenced by a large logjam in the lower portion of the reach, which blew out during several very large floods occurring between 2013 and 2015, resulting in very rapid hydrogeomorphic shifts (S5 Table).

If and how streams regain an equilibrium state following urban development is of primary interest in urban fluvial geomorphology [81]. We found evidence that some of our sites were undergoing much lower rates of hydrogeomorphic adjustment than others, which showed high rates of hydrogeomorphic change during the study period. For instance, Waterman experienced minimal change from 2011–2013, with minimal reductions in slope and incision ratio (S5 Table). Conversely, Slate and Rush appeared to continue adjusting throughout the study period, though in opposite directions: Rush widened and incised (increasing bankfull width, slope, and shear stress) while Slate aggraded (no change in bankfull dimensions, but a decrease in slope and incision ratio; S5 Table). Some studies have found that urban-induced channel enlargement tends to stabilize with time following development (e.g., [94]), and even some channels with very intense urban development can restabilize within 1–2 decades of cessation of development (e.g., [95]).

However, not all streams regain physical equilibrium [81]. Position within catchments [96, 97], erosive resistance, vegetation and substrate characteristics, and pre-development sediment transport and geomorphic characteristics [95, 98] can mediate re-equilibration. The timing and magnitude of development appear to be of little predictive value in determining those streams that will regain a geomorphic equilibrium [95]. MacFarland [47] previously found (in a subset of 10 reaches of the same study reaches as the current study) that, while timing of development was useful in some models predicting hydrogeomorphic equilibrium status, timing alone was not a strong predictor without including land-use characteristics (e.g., total catchment development). Consistent with this observation, we found no significant relationships between timing of development and the hydrogeomorphic characteristics considered here (Table 5).

#### Hydrogeomorphic-fish relationships

Many hydrogeomorphic variables have been found to correlate with fish assemblage structure and function in studies involving one-time sampling [39, 99, 100], but few (e.g., [42, 43]), if any, studies have considered paired hydrogeomorphic-fish changes over time. We observed that both individual species and assemblage composition were linked to hydrogeomorphic features in complex, unexpected, and not always consistent, ways over the study period. Fish assemblage Δ% generalist and Δ% tolerant were related to several hydrogeomorphic characteristics (e.g., incision ratio, D_50_). These and other hydrogeomorphic features are symptomatic of both urban channel structure as well as the underlying functional changes in physical processes acting at a variety of scales throughout an urbanized basin [93, 101].

In general, fish assemblages exhibited relatively low species richness and diversity across our study reaches, with large proportions of highly tolerant, generalist species (i.e., Creek Chubs, *Semolitus atromaculatus*). Insectivores were most commonly tolerant mid-water insectivores (i.e., Green Sunfish, *Lepomis cyanellus*), in line with similar studies of urban streams in temperate climates [43, 102]. Our prediction that limited floodplain access in general would lead to an increase in % trophic generalists was not well supported by our models (Table 4), although we did observe several relationships between specific fish species and hydrogeomorphic characteristics associated with floodplain access (e.g., width-of-floodprone area, width:depth ratio, incision ratio; Fig 5). Δ% generalists, however, trended with bed characteristics (i.e., fine sediment was associated with an increase in % generalists over time; Table 4), a finding consistent with other stream studies [37, 40, 41] that emphasizes the importance of a variety of factors in influencing fish assemblage composition over time. Our hypothesis that as urban-associated geomorphic alterations increased, fish density would decrease as habitat heterogeneity is lost was not supported, with riparian canopy as well as hydrologic variability (e.g., discharge) appearing in well-supported models (Table 4). These results, in concert, suggest that fish assemblages may respond indirectly to hydrogeomorphic alteration as it impacts food and habitat resources [40].

Nutrient concentrations and chemical water-quality can also have strong effects on stream fish assemblages [34, 103, 104] but were not a salient driver of fish responses in our study system (Table 4). Direct impacts of water chemistry and nutrient concentrations may be relatively subtle compared to overwhelming impacts of land use and hydrogeomorphic characteristics on habitat availability and food resources in severely altered urban stream systems [105, 106]. However, despite evidence that interannual water-quality variability is relatively low at our study reaches (S2 Fig), our coarse-level analysis limits the inferences we can draw from these results.

Hydromorphic features were also related to the distribution of individual species across our study sites. Streams in developed catchments are typified by low habitat diversity (i.e., homogeneous depth, bottom type, flow), which in turn leads to high seasonal instability in habitat and, as a result, fish assemblage characteristics [107]. These deeper, wider, more homogeneous urban channels also experience much more rapid geomorphic changes stemming from hydrologically driven reductions in channel stability [101]. Hydrologically unstable streams typically host fewer specialist species, with more habitat generalist and siltation-tolerant species [108]. Hydrogeomorphic instability driven by anthropogenic landscape alteration may favor some tolerant species that can better cope with rapid rates of environmental change [109], whereas species with more flexible feeding strategies (i.e., ability to switch food resources, even to non-optimal sources) are best able to cope with varied food resource availability [110]. This may have been (among other causes) reflected in CCA relationships in which tolerant generalist species (e.g., Creek Chub) occurred in similar relative abundances across sites that displayed a range of hydrogeomorphic characteristics.

Variability in fish assemblages across years could also be evidence of functional replacement [111], particularly relative to the partial replacement of foraging specialists by highly tolerant mid-water insectivores (e.g., Green Sunfish) or the replacement of more sensitive darter species (e.g., *Etheostoma blennioides*) by more tolerant darter species (e.g., *Etheostoma nigrum*). These replacements would not be instantaneous and may be evidenced by interannual variability in both the compositional and functional characteristics observed in our study streams. For example, Jefferson hosted one darter species in 2011 and none in 2012 or 2013, but an increasing relative abundance of midwater insectivores such as Green Sunfish (S11 and S12 Tables) suggesting a lag time following the loss of sensitive darter species before more tolerant insectivores (e.g., Green Sunfish) replaced them as the dominant insectivore taxa.

More subtle, species-specific shifts may have also been mediated by hydrogeomorphic alterations, as shown in annual variations in species-specific relationships with hydrogeomorphic features observed in canonical correspondence analysis. Johnny Darters, which are a fairly tolerant insectivorous darter species [65, 79] displayed a positive relationship with sinuosity in 2011, though a negative relationship with discharge, entrenchment ratio, and width-of-floodprone area in 2012, and no notable relationship with hydrogeomorphic predictors in 2013 (Fig 5). Sinuosity can allow increased stream-floodplain interaction via increased edge-to-area ratio [112, 113], which may have modulated energetic resources in 2011. In 2012, increased bankfull discharge, which is common in urbanized channels [114, 115] and may indicate hydraulic stress, may have been more influential due to exceptionally high spring precipitation that year [116, 117]. Study reaches with high entrenchment ratios may have also been more stressed by these large floods, as streams with limited floodplain access have a decreased ability to distribute floodwaters, thereby decreasing stream power [118]. A suite of insectivores (shifting in specific composition by year) were positively associated with incision ratio in 2011 and 2012, yet negatively associated with this characteristic in 2013 (Fig 5). Incision ratio may have indicated several alterations that may vary in their importance to insectivores. For instance, an incised reach may host an altered assemblage of aquatic macroinvertebrates [104, 119] in terms of body size and behavior that may differentially favor insectivorous fish species based on their gape and foraging habits (e.g., a denser community of smaller, more sessile macroinvertebrates may not be as highly utilized by larger, sight-foraging species such as Largemouth Bass as would larger, more mobile macroinvertebrates). Alternatively, higher incision ratios (as a result of urban land use) may reduce or alter reciprocal energy flows between streams and riparian zones [120, 121], moderating insectivorous fish species abundances through altered abundance or character of terrestrial arthropod contributions.

#### Synthesis and conclusions

Our study reaches were selected along a temporal continuum of hydrogeomorphic adjustment (as discussed above), with some sites having been developed for >100 years and others undergoing a rural-to-suburban transition several years before or during our study period. However, timing of development had no effect on fish assemblages (Table 5). In contrast, the significant negative relationships between % catchment imperviousness and both Δ fish diversity (*H’*) and D_50_ imply that certain landscape features may spatially and temporally integrate the ecological effects of urbanization on streams [14, 85]. Hydrogeomorphically-mediated habitat alterations likely underlie the observed relationship between D_50_ and fish assemblage characteristics observed here.

We observed high interannual temporal variability in hydrogeomorphic and fish-assemblage characteristics, with few variables consistently exhibiting change over time (though % tolerant individuals did increase [not evaluated statistically] over time; Figs 3 and 4). This variability was accompanied by high spatial variability. Nearly all fish assemblage characteristics varied greatly between sites, most notably diversity (*H’*) and species richness (*S*; S5 Table). This high spatiotemporal variability in individual eco-hydrogeomorphic characteristics fed into high variability in the strength and nature of relationships between fish responses and hydrogeomorphic predictors, both at the species and community levels. Though we found strong evidence that fish assemblages reflected changes in hydrogeomorphic features, conclusions regarding predicted shifts in fish assemblage characteristics (e.g., density) in relation to rates and magnitudes of hydrogeomorphic change are less certain due to the diversity of hydrogeomorphic responses observed between sites.

The need to monitor fluvial geomorphic change over time has been recognized for decades [122, 123]. However, pairing hydrogeomorphic-biotic responses over time is not common. Here, we observed a suite of relationships between fish assemblage structure and function and hydrogeomorphic features over time, suggesting that hydrogeomorphic features may be a good aggregator of land-use impacts in urban areas [93], and thus allowing understanding of cumulative impacts of multiple anthropogenic drivers of physical stream impairment. These relationships exhibited high spatiotemporal variability across our 3–5 year study period, with both hydrogeomorphic and fish assemblage characteristics varying individually as well as covarying. Overall, our work suggests that catchment landscape alteration and activities (i.e., construction) effectively cascade through a series of linkages to alter ecological function of streams (e.g., [43]), supporting an ecohydrogeomorphic approach, emphasizing hydromorphic processes, as advocated by many [44, 124, 125]. Future work on biotic-hydrogeomorphic changes should would benefit from experimental designs (e.g., factorial) that allow for more sophisticated testing of multistressor biotic responses to environmental alterations over time [126].

Management decisions can be best informed by an understanding of those hydrogeomorphic factors that are most useful to monitor in restoration activities and at what spatiotemporal scales these factors are most relevant [124]. For example, in our system, bed characteristics as well as hydrologic variability were both strongly tied to fish assemblage composition, have potential implications for ecosystem functioning, and are relatively easy to monitor over time. Based on findings from this study, we advocate integrating long-term hydrogeomorphic surveys as a part of standard stream monitoring in urban streams, which can be used for both direct applications to channel management and fish conservation.

## Supporting information

S1 Table Equations for hydrogeomorphic predictors. Equations/measurements and utility of fluvial geomorphic variables included in analyses. Note: n = Manning’s roughness = 0.05 D_50_^1/6^; for more detail (see [57]).
(TIF)

S2 Table Equations for fish response variables. Equations and utility of fish assemblage variables included in analyses.
(TIF)

S3 Table Fluvial geomorphic summary statistics by site. Mean values of fluvial geomorphic variables for all Columbus, Ohio study sites from 2011–2013 (plus 2015 for Big, Leeds, Rush, and Slate). * = second-order fluvial geomorphic variable calculated with Reference Reach Spreadsheet [60].
(TIF)

S4 Table Results from ANOVA and linear contrasts. ANOVA and linear contrasts for all fish and fluvial geomorphic variables from 2011–2013.
(TIF)

S5 Table Fish assemblage and hydrogeomorphic Δ values by site. Mean change values (Δ = 2013 value– 2011 value) for hydrogeomorphic and fish-assemblage variables from all Columbus, Ohio study sites from 2011–2013 (plus 2015 for Big, Leeds, Rush, and Slate).
(TIF)

S6 Table Chemical and nutrient water-quality data. Chemical and nutrient water-quality data for each study reach.
(TIF)

S7 Table Fish assemblage variable summary statistics by site. Mean values for fish-assemblage variables from all Columbus, Ohio study sites from 2011–2013 (plus 2015 for Big, Leeds, Rush, and Slate).
(TIF)

S8 Table Descriptive statistics for change (Δ) values. Descriptive statistics for change values (Δ = 2013 value– 2011 value) of response variables included in the analyses, including mean, standard deviation (SD), minima (min), median, and maxima (max) for each variable.
(TIF)

S9 Table Linear models for influences of hydrogeomorphic features on fish assemblage characteristics. ΔAICc = AICc (lowest)–AICc (model x).
Model selection results for eight models: (1) year only; (2) riparian canopy + year; (3) floodplain connectivity (width-of-floodprone area, sinuosity, incision ratio, width:depth ratio) + year; (4) hydrologic variability (discharge) + year; (5) bed characteristics (D_50_, slope) + year.; (6) “floodplain connectivity + year + riparian canopy” (width-of-floodprone area, sinuosity, incision ratio, width:depth ratio, year, riparian canopy cover); (7) “hydrologic variability + year + riparian canopy” (discharge, year, riparian canopy cover); and (8) “bed characteristics + year + riparian canopy” (D_50_, slope, year, riparian canopy cover). Gray shading indicates best model (lowest AICc, or, if not “year-only” model, ΔAICc ≤ 2) as well as individual hydrogeomorphic predictors showing significant or trending relationships with fish assemblage characteristics.
(TIF)

S10 Table Principal component analysis results for water quality variables. Loading and *r*^*2*^ for each water quality variable, and eigenvalue and variance for the first two principal components of the water quality principal component analysis.
(TIF)

S11 Table Data for fish assemblage characteristics for each sampling time for each study reach. (TIF)

S12 Table Relative abundances of fish species by site and year. (TIF)

S13 Table Data for hydrogeomorphic characteristics for each sampling time for each study reach. (TIF)

S1 Fig Hydrogeomorphic principal component analysis. Principal component analysis (PCA) loading plot for hydrogeomorphic characteristics for all reaches and years sampled.
(TIF)

S2 Fig Boxplots showing average chemical water quality and nutrient data +/- 1 SE for all study reaches. (a) total nitrogen (mg L-1); (b) total phosphorus (mg L-1); (c) total Hg (ppt); (d) TDS (mg L-1) using samples collected in 2014, 2016, 2017, and 2018.
(TIF)

S3 Fig Line graphs of hydrogeomorphic predictors by year. Average values +/- 1 SE for the ten hydrogeomorphic variables examined at the 12 study sites from 2011–2013, and in 2015 (four sites only): (a) width-of-floodprone area (m), (b) shear stress (kPa), (c) entrenchment ratio, (d) bankfull width (m), (e) width:depth ratio, (f) discharge (m^3^ s^-1^), (g) incision ratio, (h) sinuosity, (i) D_50_ (mm), and (j) slope (%). Gray bars indicate change in scale as 2015 values were much more variable than 2011–2013 values for a particular variable.
(TIF)

S4 Fig Line graphs of fish assemblage variables by year. Average values +/- 1 SE for fish assemblages at the 12 study reaches from 2011–2013, and in 2015 (four sites only: (a) % tolerant, (b) % trophic generalists, (c) diversity (*H’*), (d) no. darter species, (e) density (no. m^-3^), (f) species richness (*S*), (g) % herbivores.
(TIF)
