# Supplementary figures and images for: Coupled fish-hydrogeomorphic responses to urbanization in streams of Columbus, Ohio, USA
Source: PLoS One. 2020 Jun 15;15(6):e0234303. doi: 10.1371/journal.pone.0234303 (PMC7295201; doi:10.1371/journal.pone.0234303)

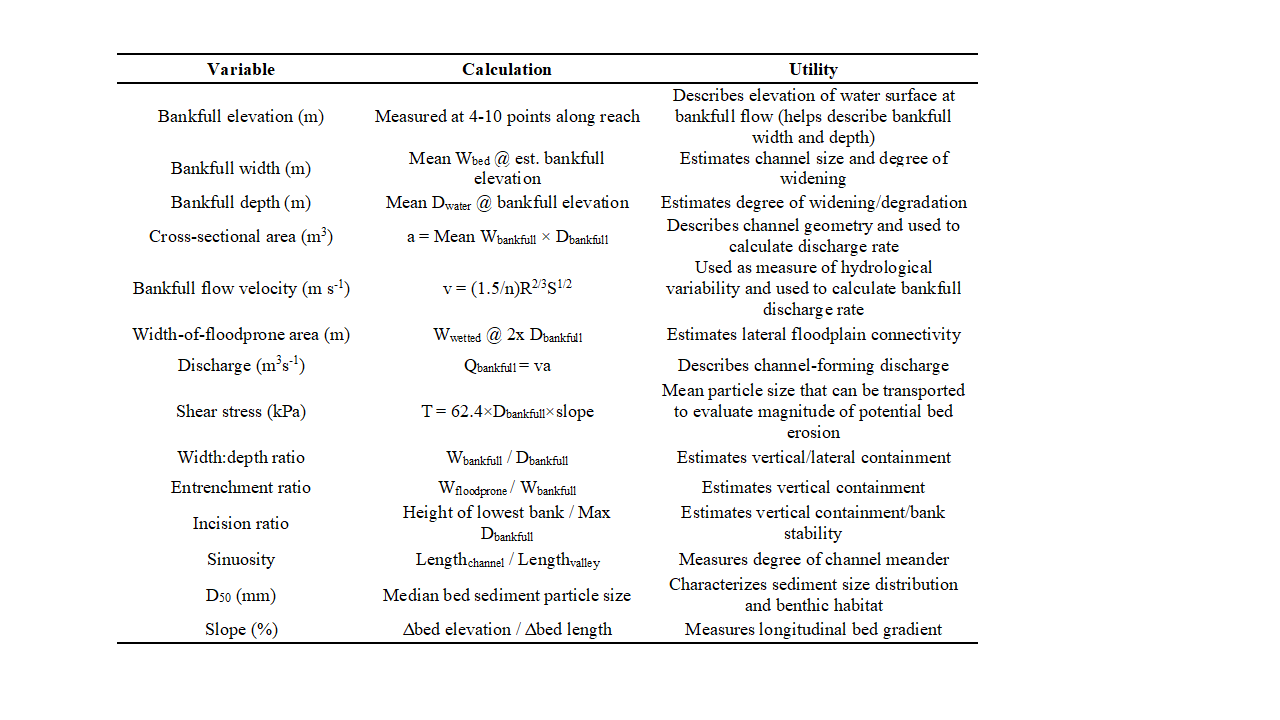

Supplement: S1 Table — Equations/measurements and utility of fluvial geomorphic variables included in analyses. Note: n = Manning’s roughness = 0.05 D501/6; for more detail (see [57]). (TIF) [file pone.0234303.s001.tif]

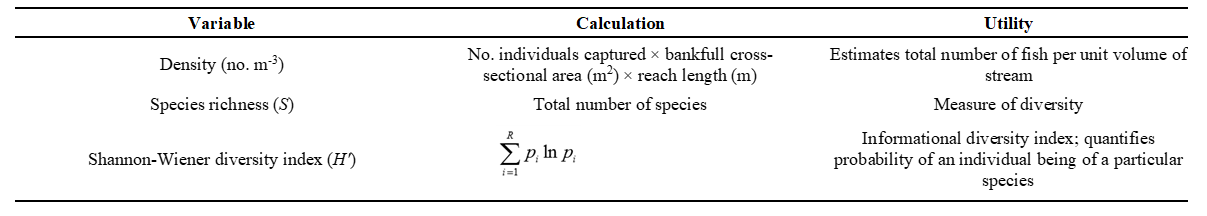

Supplement: S2 Table — Equations and utility of fish assemblage variables included in analyses. (TIF) [file pone.0234303.s002.tif]

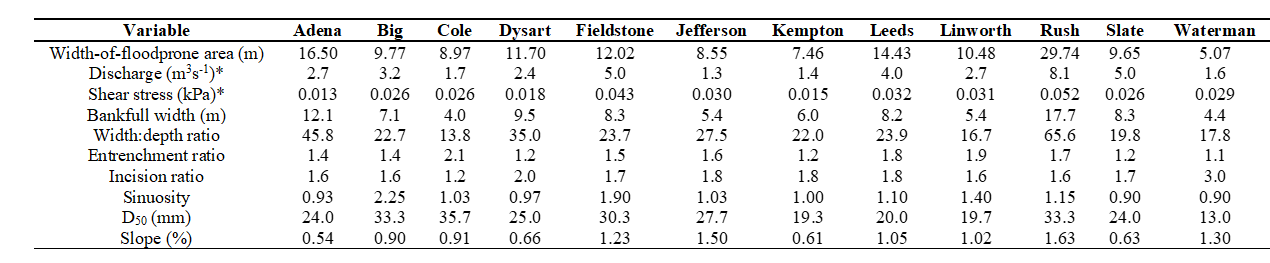

Supplement: S3 Table — Mean values of fluvial geomorphic variables for all Columbus, Ohio study sites from 2011–2013 (plus 2015 for Big, Leeds, Rush, and Slate). * = second-order fluvial geomorphic variable calculated with Reference Reach Spreadsheet [60]. (TIF) [file pone.0234303.s003.tif]

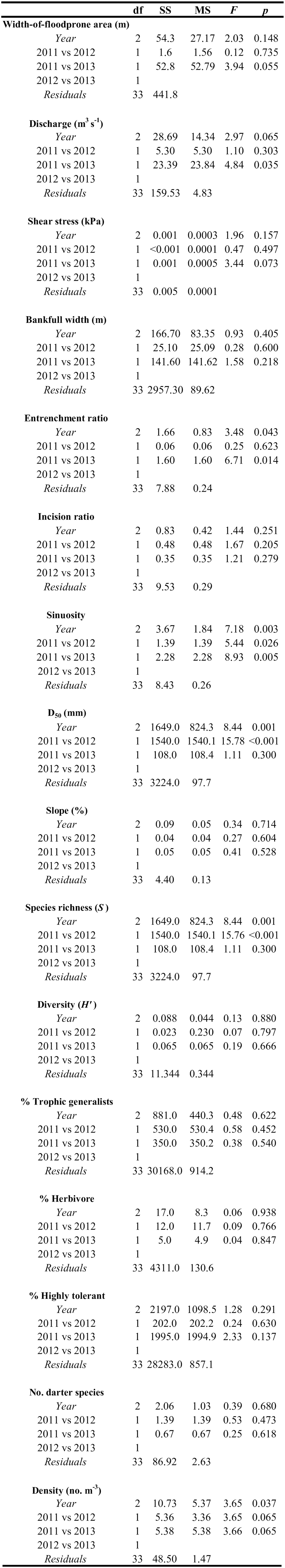

Supplement: S4 Table — ANOVA and linear contrasts for all fish and fluvial geomorphic variables from 2011–2013. (TIF) [file pone.0234303.s004.tif]

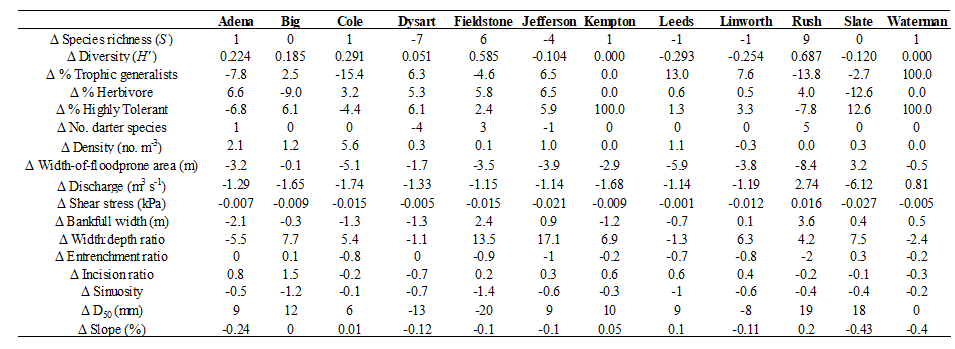

Supplement: S5 Table — Mean change values (Δ = 2013 value– 2011 value) for hydrogeomorphic and fish-assemblage variables from all Columbus, Ohio study sites from 2011–2013 (plus 2015 for Big, Leeds, Rush, and Slate). (TIF) [file pone.0234303.s005.tif]

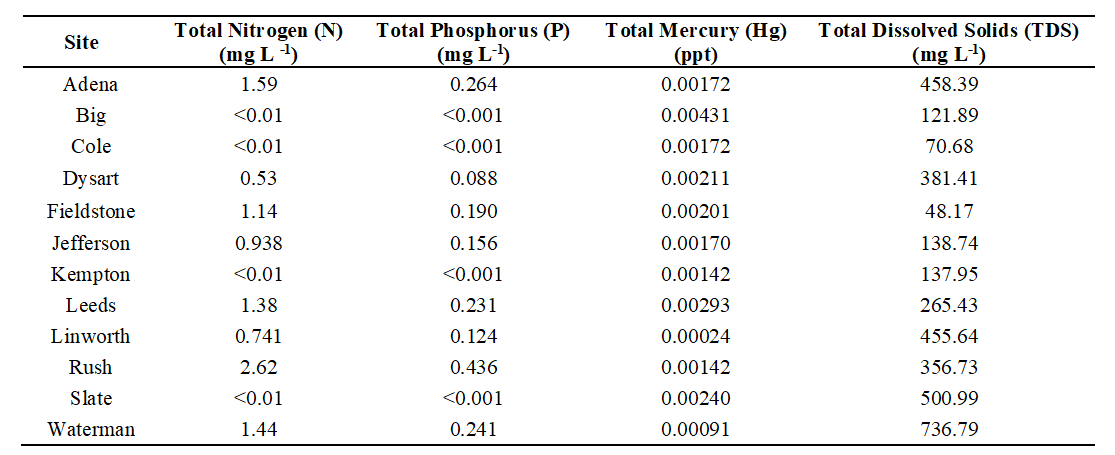

Supplement: S6 Table — Chemical and nutrient water-quality data for each study reach. (TIF) [file pone.0234303.s006.tif]

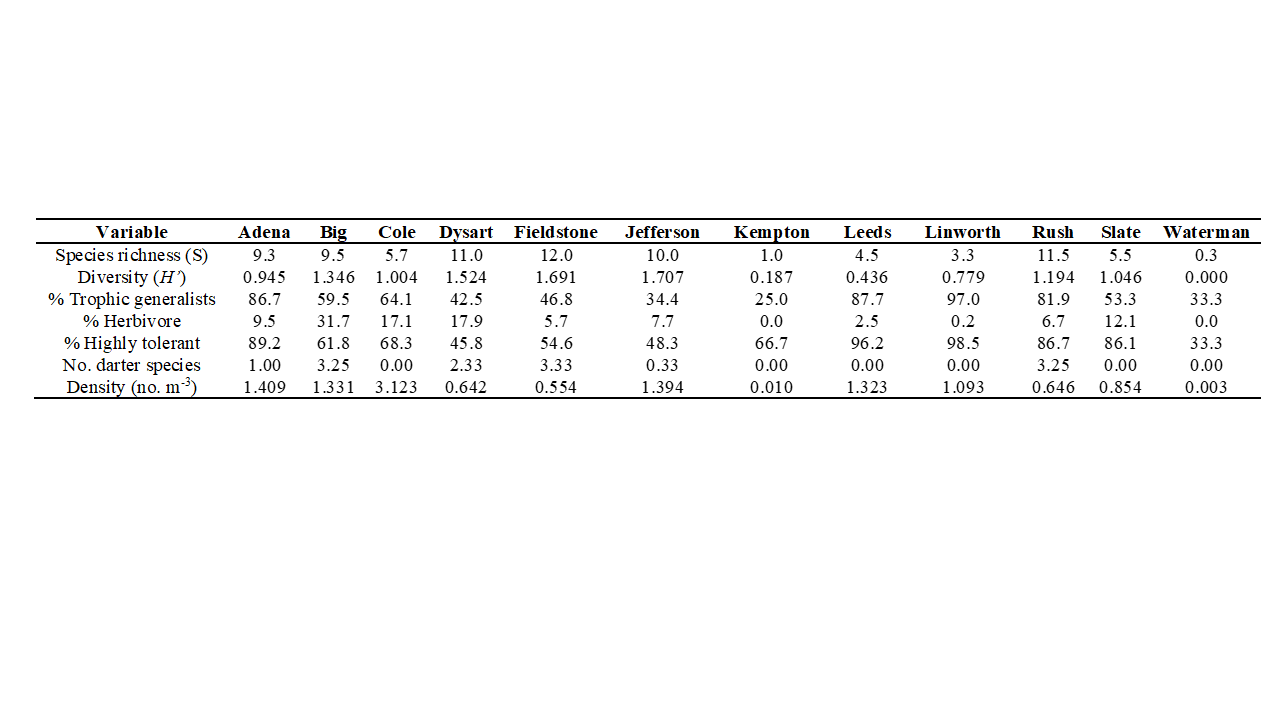

Supplement: S7 Table — Mean values for fish-assemblage variables from all Columbus, Ohio study sites from 2011–2013 (plus 2015 for Big, Leeds, Rush, and Slate). (TIF) [file pone.0234303.s007.tif]

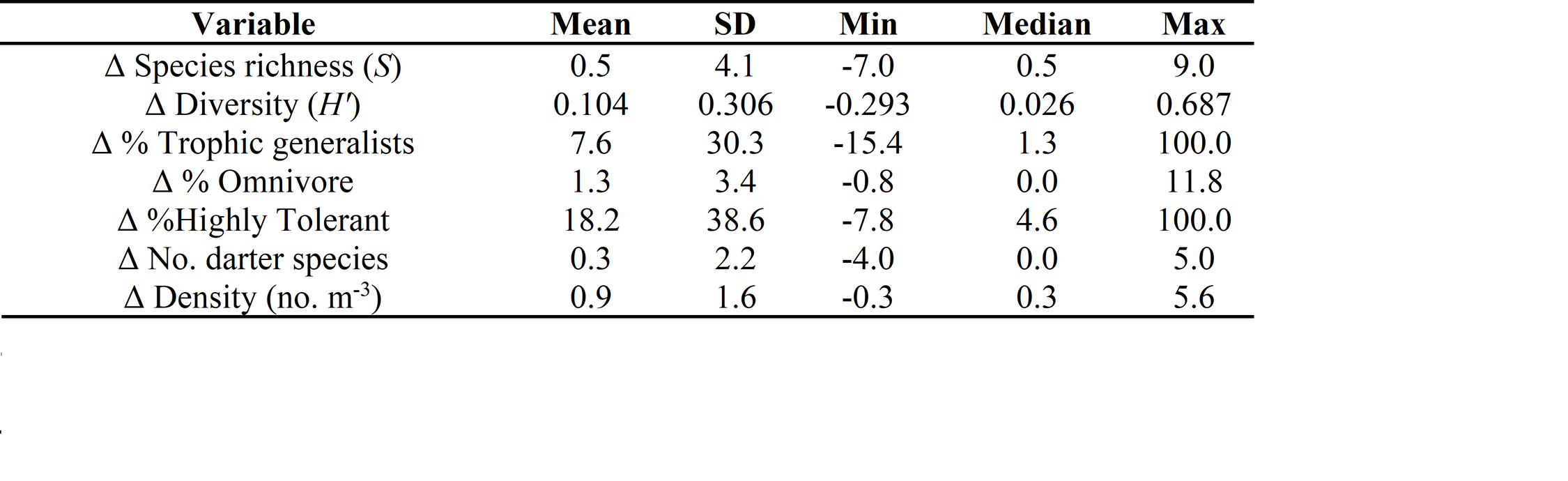

Supplement: S8 Table — Descriptive statistics for change values (Δ = 2013 value– 2011 value) of response variables included in the analyses, including mean, standard deviation (SD), minima (min), median, and maxima (max) for each variable. (TIF) [file pone.0234303.s008.tif]

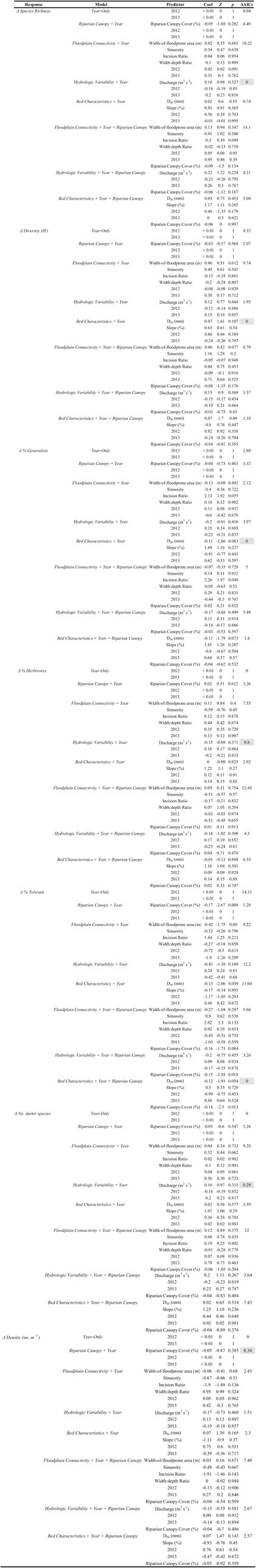

Supplement: S9 Table — ΔAICc = AICc (lowest)–AICc (model x). Model selection results for eight models: (1) year only; (2) riparian canopy + year; (3) floodplain connectivity (width-of-floodprone area, sinuosity, incision ratio, width:depth ratio) + year; (4) hydrologic variability (discharge) + year; (5) bed characteristics (D50, slope) + year.; (6) “floodplain connectivity + year + riparian canopy” (width-of-floodprone area, sinuosity, incision ratio, width:depth ratio, year, riparian canopy cover); (7) “hydrologic variability + year + riparian canopy” (discharge, year, riparian canopy cover); and (8) “bed characteristics + year + riparian canopy” (D50, slope, year, riparian canopy cover). Gray shading indicates best model (lowest AICc, or, if not “year-only” model, ΔAICc ≤ 2) as well as individual hydrogeomorphic predictors showing significant or trending relationships with fish assemblage characteristics. (TIF) [file pone.0234303.s009.tif]

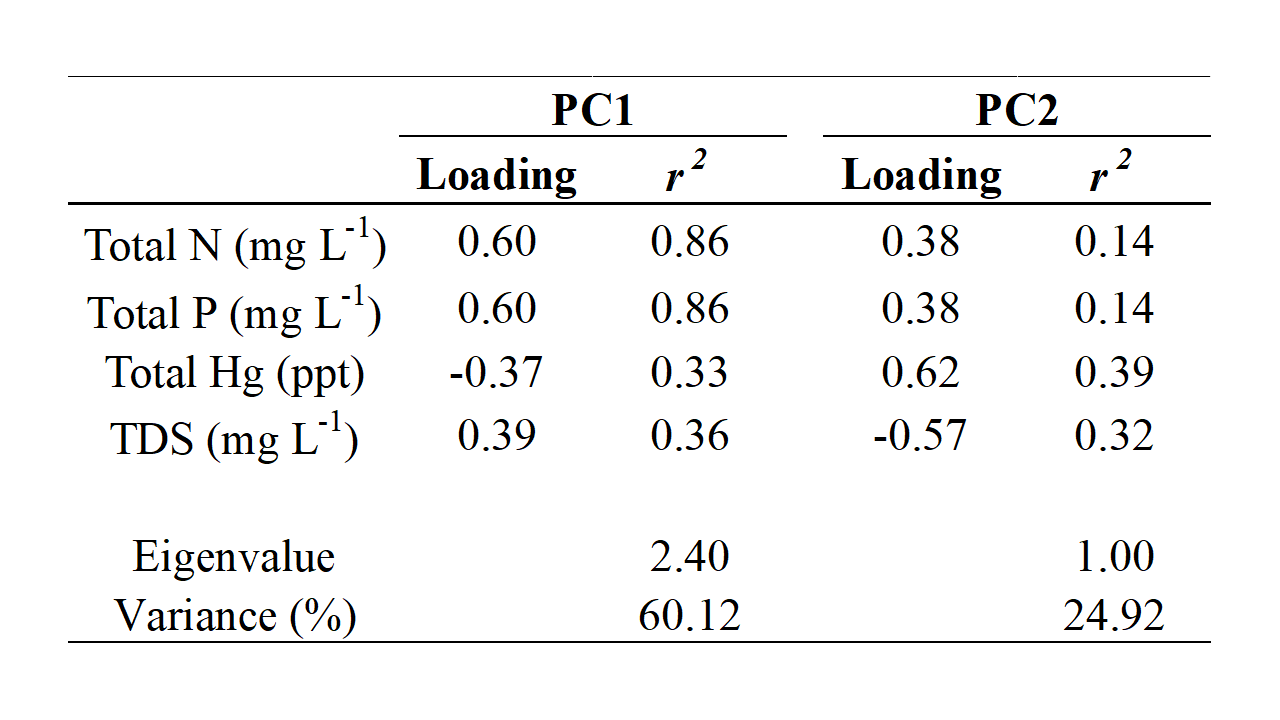

Supplement: S10 Table — Loading and r2 for each water quality variable, and eigenvalue and variance for the first two principal components of the water quality principal component analysis. (TIF) [file pone.0234303.s010.tif]

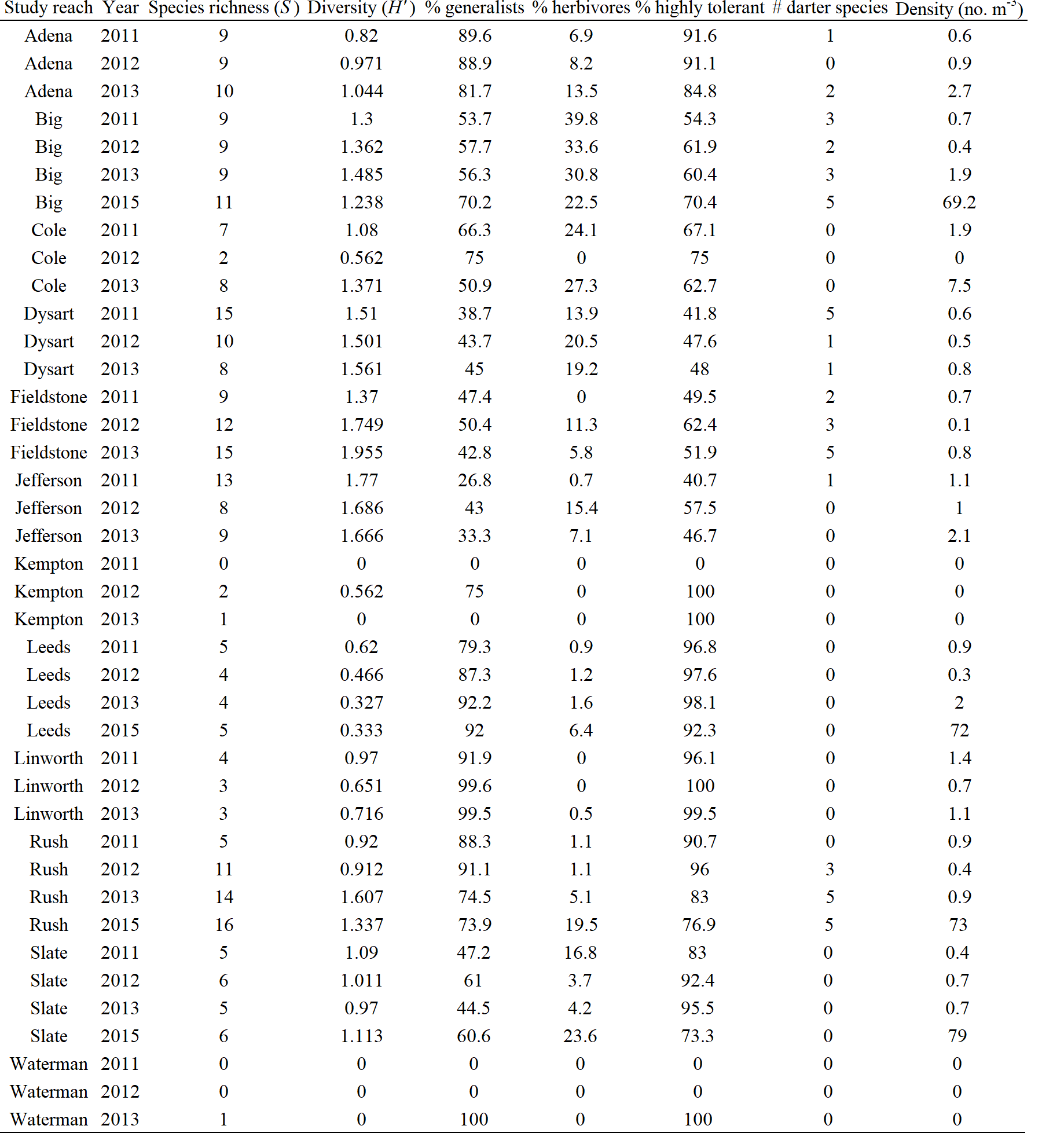

Supplement: S11 Table — (TIF) [file pone.0234303.s011.tif]

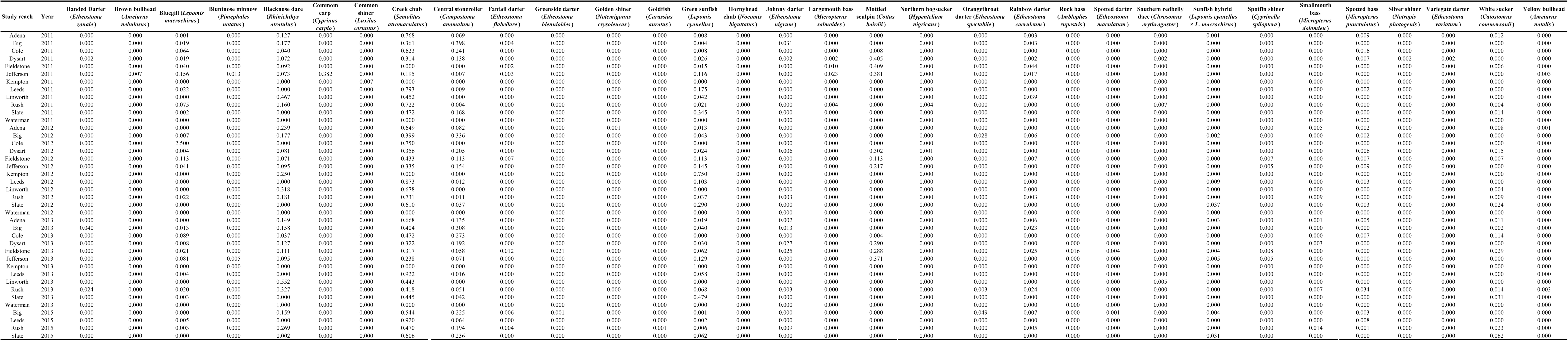

Supplement: S12 Table — (TIF) [file pone.0234303.s012.tif]

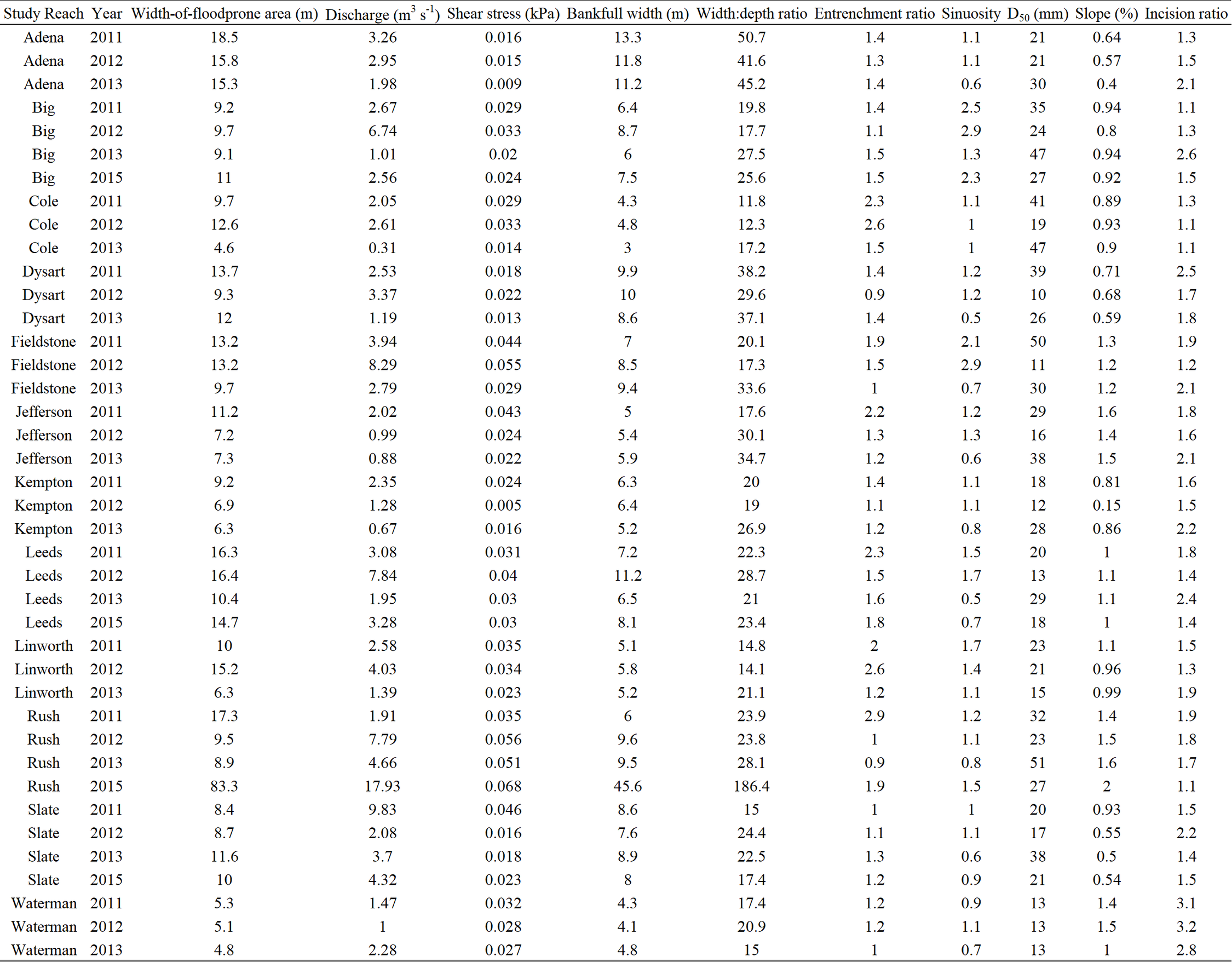

Supplement: S13 Table — (TIF) [file pone.0234303.s013.tif]

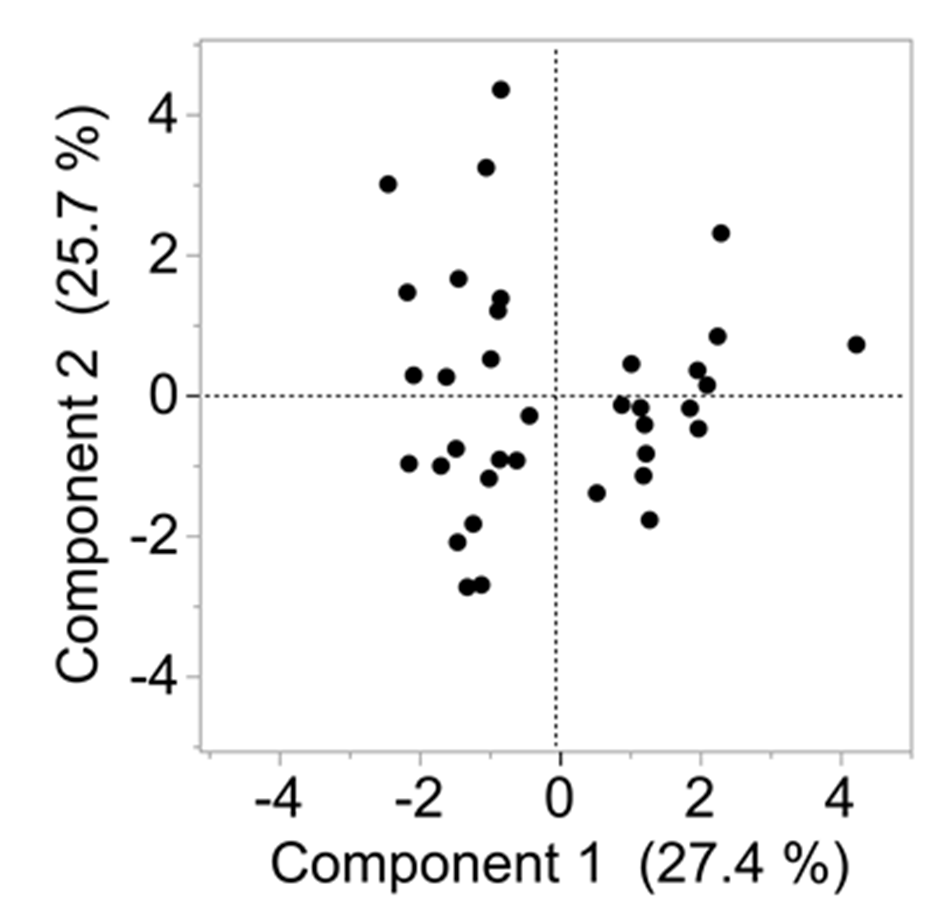

Supplement: S1 Fig — Principal component analysis (PCA) loading plot for hydrogeomorphic characteristics for all reaches and years sampled. (TIF) [file pone.0234303.s014.tif]

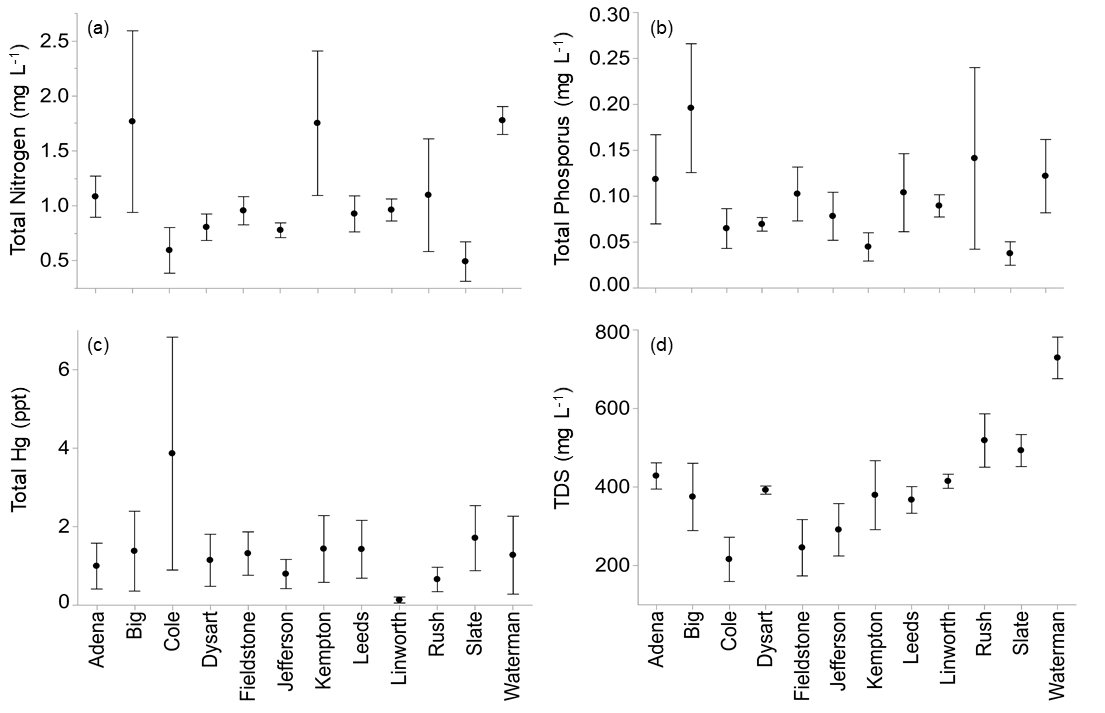

Supplement: S2 Fig — (a) total nitrogen (mg L-1); (b) total phosphorus (mg L-1); (c) total Hg (ppt); (d) TDS (mg L-1) using samples collected in 2014, 2016, 2017, and 2018. (TIF) [file pone.0234303.s015.tif]

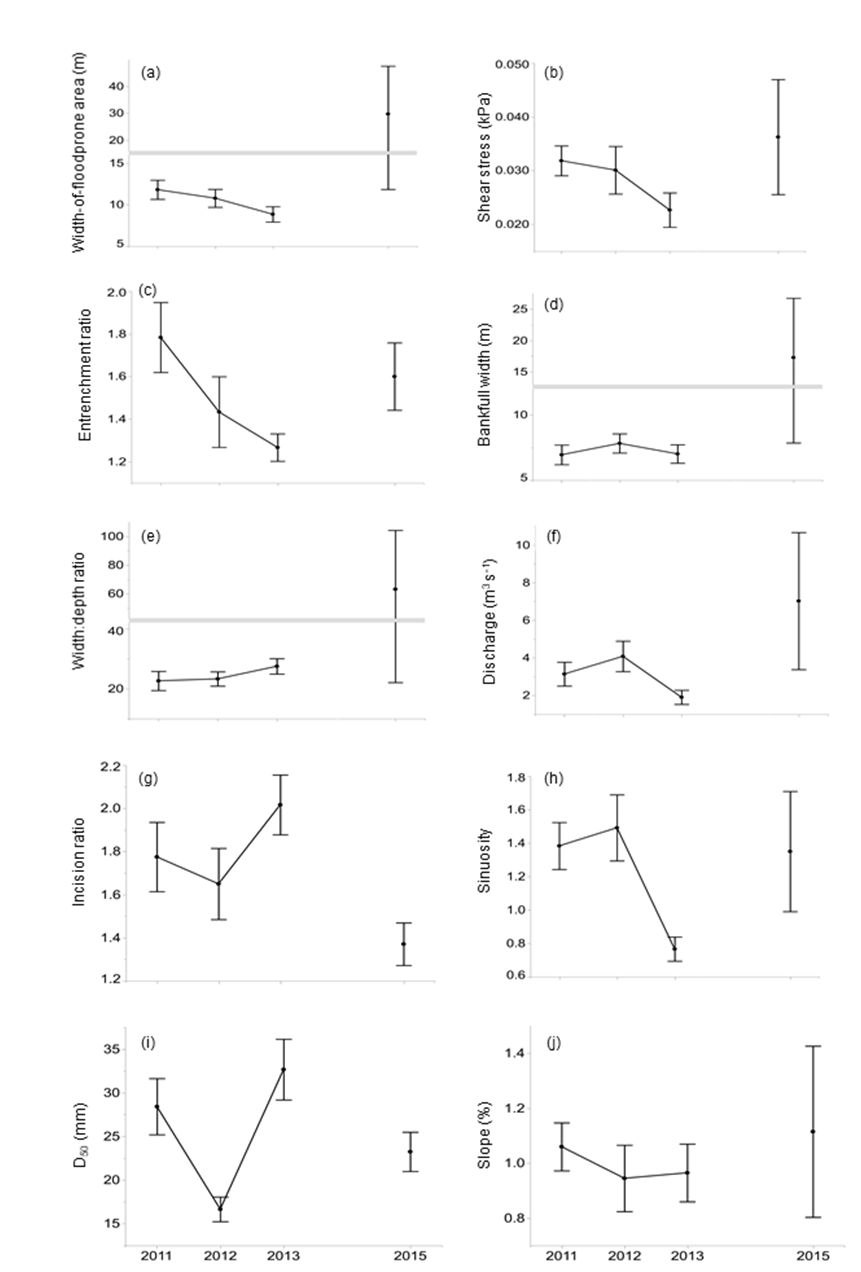

Supplement: S3 Fig — Average values +/- 1 SE for the ten hydrogeomorphic variables examined at the 12 study sites from 2011–2013, and in 2015 (four sites only): (a) width-of-floodprone area (m), (b) shear stress (kPa), (c) entrenchment ratio, (d) bankfull width (m), (e) width:depth ratio, (f) discharge (m3 s-1), (g) incision ratio, (h) sinuosity, (i) D50 (mm), and (j) slope (%). Gray bars indicate change in scale as 2015 values were much more variable than 2011–2013 values for a particular variable. (TIF) [file pone.0234303.s016.tif]

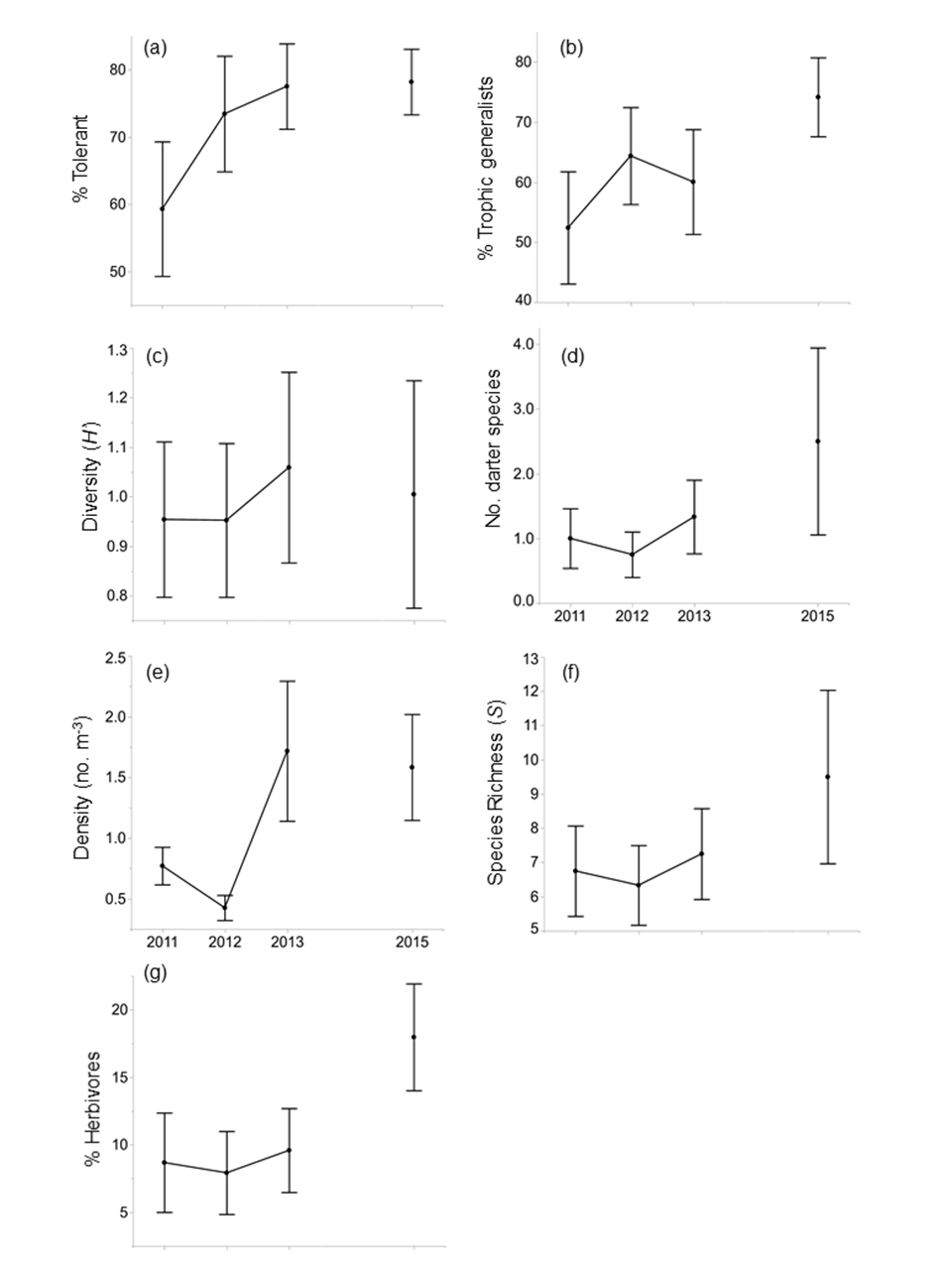

Supplement: S4 Fig — Average values +/- 1 SE for fish assemblages at the 12 study reaches from 2011–2013, and in 2015 (four sites only: (a) % tolerant, (b) % trophic generalists, (c) diversity (H’), (d) no. darter species, (e) density (no. m-3), (f) species richness (S), (g) % herbivores. (TIF) [file pone.0234303.s017.tif]
